# Fast detection of de novo copy number variants from SNP arrays for case-parent trios

**DOI:** 10.1186/1471-2105-13-330

**Published:** 2012-12-12

**Authors:** Robert B Scharpf, Terri H Beaty, Holger Schwender, Samuel G Younkin, Alan F Scott, Ingo Ruczinski

**Affiliations:** 1 Department of Oncology, Johns Hopkins University, Baltimore, MD, USA; 2 Department of Epidemiology, Johns Hopkins Bloomberg School of Public Health, Baltimore, MD, USA; 3 Mathematical Institute, Heinrich-Heine-University Düsseldorf, 40225 Düsseldorf, Germany; 4 Department of Medicine, Johns Hopkins University, Baltimore, MD, USA; 5 Department of Biostatistics, Johns Hopkins Bloomberg School of Public Health, Baltimore, MD, USA

**Keywords:** Trios, Oral cleft, Copy number variants, de novo, High-throughput arrays, Segmentation, batch effects, Genomic waves

## Abstract

**Background:**

In studies of case-parent trios, we define copy number variants (CNVs) in the offspring that differ from the parental copy numbers as de novo and of interest for their potential functional role in disease. Among the leading array-based methods for discovery of de novo CNVs in case-parent trios is the joint hidden Markov model (HMM) implemented in the PennCNV software. However, the computational demands of the joint HMM are substantial and the extent to which false positive identifications occur in case-parent trios has not been well described. We evaluate these issues in a study of oral cleft case-parent trios.

**Results:**

Our analysis of the oral cleft trios reveals that genomic waves represent a substantial source of false positive identifications in the joint HMM, despite a wave-correction implementation in PennCNV. In addition, the noise of low-level summaries of relative copy number (log R ratios) is strongly associated with batch and correlated with the frequency of de novo CNV calls. Exploiting the trio design, we propose a univariate statistic for relative copy number referred to as the *minimum distance* that can reduce technical variation from probe effects and genomic waves. We use circular binary segmentation to segment the minimum distance and maximum a posteriori estimation to infer de novo CNVs from the segmented genome. Compared to PennCNV on simulated data, *MinimumDistance* identifies fewer false positives on average and is comparable to PennCNV with respect to false negatives. Genomic waves contribute to discordance of PennCNV and MinimumDistance for high coverage de novo calls, while highly concordant calls on chromosome 22 were validated by quantitative PCR. Computationally, MinimumDistance provides a nearly 8-fold increase in speed relative to the joint HMM in a study of oral cleft trios.

**Conclusions:**

Our results indicate that batch effects and genomic waves are important considerations for case-parent studies of de novo CNV, and that the minimum distance is an effective statistic for reducing technical variation contributing to false de novo discoveries. Coupled with segmentation and maximum a posteriori estimation, our algorithm compares favorably to the joint HMM with MinimumDistance being much faster.

## Background

High-throughput arrays such as array comparative genomic hybridization (aCGH) and single nucleotide polymorphism (SNP) arrays provide high resolution maps of deletions and duplications. Such maps have been used to characterize the extent of CNVs in normal populations such as HapMap [1] and to study the association of duplications and deletions in case-control study designs [2-5]. A popular alternative to the case-control is the case-parent trio design, comprised of affected offspring and unaffected parents. De novo CNVs are of particular interest in case-parent trios for their potential to have a functional role in the genesis of the disease phenotype. While numerous methods for detection of CNVs in independent samples are available, there are comparatively few statistical methods for the detection of de novo CNVs in case-parent trios. Comparisons of alternative algorithms for de novo CNV detection have been limited and the extent to which technical artifacts such as genomic waves [6,7] and batch effects [8] contribute to false positive identifications has not been well described.

Among the predominant algorithms for array-based CNV discovery are segmentation algorithms that segment the genome into regions of constant copy number [9-13] and hidden Markov models (HMMs) that simultaneously segment and classify the latent copy number. Segmentation algorithms for copy number have been extended to accommodate multi-sample inference, including segmentation of paired tumor-normals [14-16] and independent samples [17-20]. Post hoc approaches for classifying the gain or loss of copy number from segmentation methods have been proposed [21]. Similarly, HMM algorithms were originally formulated for aCGH platforms [22] and many innovations were subsequently proposed. For example, distance-based transition probabilities [6], fully Bayesian HMMs [23], reversible jump and approximate sampling Markov chain Monte Carlo (MCMC) [24,25], iterative approaches to parameter estimation [26], alternatives to the Viterbi algorithm [27], and higher order Markov chains [28]. As HMMs readily accomodate multiple data sequences, the observation that copy number can be estimated from genotyping arrays [29] led to the development of several HMMs that jointly model copy number and genotypes at SNPs [30-37].

Statistical methods for the detection of de novo CNVs in case-parent trios have evolved from two-stage models to joint models. For the former, an HMM or segmentation method is fit independently to each sample of a trio and post hoc classification is obtained by identifying non-overlapping CNV in the offspring [38] or through posterior calling algorithms that incorporate probabilistic models of Mendelian CNV transmission [31]. While HMMs and segmentation methods for the analysis of multiple samples are available [17-20,39], these approaches target the detection of recurrent CNV in independent samples as opposed to de novo CNV in related samples. Ultimately, the two-stage posterior calling algorithm led to a *joint* HMM implemented in the software PennCNV that simultaneously integrates measures of relative copy number and allele frequencies of a parent-offspring trio [40]. Throughout this paper, we refer to measures of relative copy number and allele frequencies as log R ratios and B allele frequencies, respectively, as defined previously [41]. The joint HMM outperforms the two-stage predecessor in a comparison of the two approaches [40].

In this paper, we apply a wave correction procedure [7] implemented as part of the joint HMM to a case-parent study of oral clefts. Our findings motivate an alternative marker-specific measure of relative copy number, the *minimum distance*, that directly exploits the trio design. We use a standard single-sample segmentation algorithm to segment the univariate minimum distance and maximum a posteriori estimation to infer the de novo status of each segment. We compare the *MinimumDistance* algorithm to the joint HMM on simulated data and the oral cleft study. As the discovery of de novo deletions were identified as a priority by our epidemiologic collaborators for the oral cleft study, we give particular emphasis to findings with respect to de novo deletions. The R package MinimumDistance is available from Bioconductor [42].

## Results and discussion

### Motivation

The main objective of our research is the delineation of copy number alterations present in the offspring that differ from parental copy numbers (defined as de novo), with an emphasis on false positive identifications and computational speed. We evaluate these issues on a case-parent study of 2,082 oral cleft trios.

We applied the joint HMM implemented in PennCNV with wave correction to the oral cleft trios. The analysis required an average of 130 minutes for a single trio and approximately 2.5 weeks for the oral cleft study when computation was distributed across 10 high performance nodes. Among 1,741 trios passing quality control (see Methods), the median number of de novo calls was 3 with an interquartile range of 2 to 5. To assess batch differences in the de novo call frequencies, we use the chemistry plate on which the samples were processed as a surrogate. We observed statistically significant differences by batch for the median absolute deviation (MAD) log R ratio (analysis of variance F-statistic with 76 and 4726 degrees of freedom was 25.07). While quality control removed trios for which the MAD and corresponding call frequencies were extreme, the mean MAD for each batch was positively correlated with the mean frequency of de novo deletion calls (Spearman correlation coefficient 0.54).

**Figure 1:**
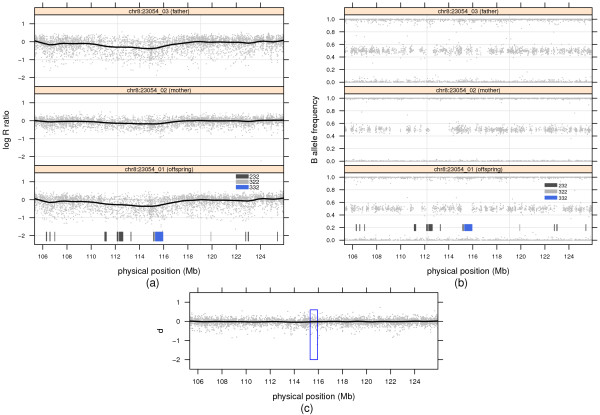
**Genomic waves appearing in parents and offspring induce false positive de novo deletions.** Panels in **(a)** and **(b)** plot log R ratios and B allele frequencies, respectively, for father (top), mother (middle), and offspring (bottom). Overlaying the log R ratio plots is a lowess curve with a span of 1/10. The calls from the joint HMM are indicated in the offspring panel of Figures (a) and (b). The state codes 232, 322, and 332 correspond to a paternally inherited deletion, a maternally inherited deletion, and de novo deletion, respectively. **(c)** The minimum distance calculated from the log R ratios in (a). Overlaying the minimum distance is a lowess curve with the same span as in (a) and (b). The blue rectangle demarcates the de novo hemizygous deletion called by PennCNV.

To identify data characteristics contributing to unusually high de novo deletion call frequencies, we plotted the log R ratios and B allele frequencies against their genomic physical position. In many trios with high de novo deletion frequencies, we observed smooth genomic waves with inferred breakpoints alternating between diploid and deletion states coinciding with regions of homozygosity. For example, a trough of approximately 5 Mb on chromosome 8 spans a 600 kb de novo deletion as well as several transmitted CNV called by PennCNV (Figure 1a). A very similar trough is evident in the father and to a lesser degree in the mother (panels 1 and 2, Figure 1a). While it appears incongruous that the copy number is not the same for the parents and offspring, regions of homozygous genotypes with corresponding B allele frequency emissions near 0 and 1 in the parents and offspring demarcate the called deletion boundaries (Figure 1b). Specifically, paternally transmitted deletions are called in regions of the trough in which both the father and offspring are homozygous, maternally transmitted deletions are called in regions of the trough in which the mother and offspring are homozygous, and a de novo deletion is called where only the offspring is homozygous. An alternative explanation for the data is that the trough is promoting a false deletion state. As heterozygous B allele frequency emissions are informative for diploid copy number, the HMM captures regions in which there are no heterozygous genotypes to oppose the deletion state that is favored by the negative log R ratios in the trough. To the extent that regions of homozygosity are common and genomic waves persist following wave correction, the de novo call frequencies may far exceed the true number of de novo CNVs. For example, the trio featured in Figure 1 has 51 de novo CNVs, or nearly 20-fold the median frequency in the oral cleft study.

### Algorithm

#### Definition of the minimum distance

Consider the difference in log R ratio (*r*) between offspring (O) and father (F) at a single marker, calculated as *r*_*O*_−*r*_*F*_. We denote the paternal distance by *δ*_*F*_. A comparable calculation for offspring and mother provides a measure of the maternal distance, denoted by *δ*_*M*_. We define the minimum distance between parents and offspring as

(1) $$d=\underset{\delta \in \{\delta_{F},\delta_{M}\}}{\text{arg min}}|\delta |.$$

The calculation is easily vectorized in R and its computation for ≈ 610,000 log R ratios obtained from Illumina’s 610 quad array for a single trio is nearly instantaneous. Denoting the minimum distance vector by ***d***, consecutive negative or positive values in a genomic interval suggest DNA copy number loss or gain, respectively, relative to the most similar parental copy number. Although its calculation at a given marker is independent of the neighboring markers, the minimum distance can reduce technical variation from correlated probe-effects as well as the peaks and troughs of genomic waves that vary smoothly over large regions of the genome (e.g., Figure 1c). Alternatives to ***d*** include the difference of the offspring log R ratios and the CNV-transmitting parent. However, such an alternative requires inference of the CNV-transmitting parent and a trade-off in variance when technical factors such as wave and probe effects in the offspring are more correlated with the non-CNV transmitting parent.

#### Segmentation of the minimum distance

Single-sample segmentation algorithms applied to the univariate ***d*** can be used to identify breakpoints of potentially de novo CNVs. We currently favor circular binary segmentation (CBS) [9,12] for its maturity in the Bioconductor package DNAcopy and its use as a benchmark in comparison papers for CNV detection algorithms [43]. Nonstandard options for CBS implemented in MinimumDistance include special handling of large gaps in the array’s coverage of the genome (see Methods) and a pruning step to remove breakpoints that is a function of the number of markers on a segment (coverage) and the standardized difference in segment means (see Additional file 1).

The minimum distance can reduce artifacts that are shared by one or both parents and the offspring. In the motiving example (Figure 1), we argue that genomic waves contribute to false de novo and transmitted deletions when the trough of a genomic wave spans regions lacking heterozygous genotypes. Application of CBS to ***d*** calculated in the motivating example smooths the trough of the genomic wave (not shown), thereby avoiding local maxima in the likelihood identified by the joint HMM. The subsequent classification of the trio copy number (discussed next) for the minimum distance segment spanning the trough overwhelmingly favors a diploid trio copy number state due to the large number of heterozygous genotypes in the broader region.

As the minimum distance is a relative measure, regions with non-zero minimum distance do not necessarily indicate de novo CNVs. For example, a 300 kb region with positive ***d*** on chromosome 14 suggests a de novo duplication (bottom panel, Additional file 2: Figure S1). However, visual inspection of the B allele frequencies and log R ratios reveals deletions in both parents while the offspring is diploid (panels 1-3, Additional file 2: Figure S1). To avoid false positive de novo CNV calls for regions such as chromosome 14, estimation of the absolute copy numbers is needed. We use maximum a posteriori estimation to infer the absolute copy numbers, as described in the following section.

#### Maximum a posteriori estimation

We classify the copy number states of the minimum distance segments using a fully probabilistic model based in part on the joint HMM. Our approach delineates de novo events by finding the mode of the distribution of P(states | data, …) over the set of possible trio states. More formally, the maximum a posteriori estimate for the trio copy number for candidate segment *l* is defined as

(2) $${\hat{s}}_{l}=\left\{\begin{array}{ll} \underset{s_{l}\in S}{\text{arg max}}P(s_{l}|B_{l},R_{l},\Theta ) & \mathrm{if}l=1\\ \underset{s_{l}\in S}{\text{arg max}}P(s_{l}|B_{l},R_{l},s_{l-1},\Theta ) & \mathrm{if}l>1. \end{array}\right)$$

The vector ***s***_*l *_contains the copy number state symbols for the trio denoted as *xyz*, where *x* is the state symbol for the father, *y* is the state symbol for the mother, and *z* is the state symbol for the offspring. The copy number state symbols are 1 = homozygous deletion, 2 = hemizygous deletion, 3 = diploid copy number, 5 = single copy gain, and 6=two copy gain. The triplet 332, for example, corresponds to a de novo hemizygous deletion in the offspring. These integer state symbols are used to be consistent with PennCNV, and are subject to change in the software implementation of MinimumDistance. The set of 121 biologically plausible trio copy number states is denoted by *S*, and excludes 4 of the 5^3^ possible combinations of trio states in which the parents are both homozygous null and the offspring has one or more copies. The parameter **Θ** denotes other parameters for our model, including the transition probabilities and initial state probabilities. The matrices of B allele frequencies (***B***_*l*_) and log R ratios (***R***_*l*_) are *n*_*l *_× 3 matrices where *n*_*l*_ is the number of markers spanned by the segment *l* (hereafter, referred to as coverage) and columns are individuals in the trio. We remark that the ratio of $P({\hat{s}}_{l}|B_{l},R_{l},\Theta )$ to the probability of a trio of diploid copy numbers can be used to rank de novo CNVs.

The conditional probability of the trio copy number in equation (2) can be re-expressed using Bayes’ rule as a product of the likelihood and the joint probability of the copy number states. (Hereafter, we refer to the conditional probability in equation (2) as a posterior probability.) Factoring the joint probability of the trio state as in Wang *et al.*[40], we write the posterior probability as

(3) $$\begin{array}{lll} \;P(S_{1}|B_{1},R_{1},\Theta ) & \propto P(B_{1},R_{1}|S_{1},\Theta )P(s_{1,O}|s_{1,F},s_{1,M},\Theta )\; & \;\\ & \;\times \underset{k\in \{F,M\}}{\prod }P(s_{1,k}|\Theta )\; \end{array}$$

for the first segment and

(4) $$\begin{array}{lll} \;P(s_{l}|B_{l},R_{l},s_{l-1},\Theta ) & \propto P(B_{l},R_{l}|s_{l},\Theta )\; & \;\\ & \;\times P(s_{l,O},s_{l-1,O}|s_{l,F},s_{l-1,F},s_{l,M},s_{l-1,M},\Theta )\;\\ & \;\times \underset{k\in \{F,M\}}{\prod }P(s_{l,k}|s_{l-1,k},\Theta )P(s_{l-1,k}|\Theta )\; & \; \end{array}$$

for segments *l *> 1. This is a first order Markov model incorporating terms P(*s*_1,*O*_|…) and P(*s*_*l*,*O*_*s*_*l*−1,*O*_|…) for Mendelian transmission of CNVs as implemented in the joint HMM [40], but assessed on previously determined DNA segments (see Methods). Assuming conditional independence of the log R ratios and B allele frequencies given the unobserved copy number states, the likelihood is

$$P(B_{l},R_{l}|s_{l},\Theta )=\underset{k\in \{F,M,O\}}{\prod }\underset{i\in l}{\prod }P(r_{i,k}|s_{l},\Theta )P(b_{i,k}|s_{l},\Theta ).$$

As copy number estimates from hybridization-based arrays are noisy, our goal is to estimate the likelihood robustly.

Our approach for robust-to-outlier estimation of a sample’s log R ratio likelihood is predicated on a mixture distribution for the emitted log R ratios. Specifically, for individual k of a trio and marker i, we assume a mixture distribution for the log R ratio given by

(5) $$\begin{array}{lll} r_{i,k}|s,\mu_{r,k,s},\sigma_{r,k,s},\varepsilon_{r,k} & ∼(1-\varepsilon_{r,k})N(r_{i,k}|\mu_{r,k,s},\sigma_{r,k,s})\;\\ & \;+\varepsilon_{r,k}\;U(r_{i,k}|l_{r},u_{r}),\; & \; \end{array}$$

where the normal component captures within-sample variation for copy number state *s* and the uniform component captures outliers arising from technical artifacts that we assume to be independent of the latent copy number. The parameter *ε*_*r*,*k*_ is the probability of observing an outlier log R ratio in sample *k*. Similar mixture models have been proposed for aCGH [44], and adapted here for genotyping platforms. Estimation of the parameters for the means, variances, and outlier mixture probabilities is carried out via the Baum-Welch algorithm as described in the Methods section [45].

With the exception of the homozygous null state, robust-to-outlier estimation of the B allele frequency likelihood for a sample is also implemented via a mixture model. In particular, for positive copy number states we assume a theoretical mixture distribution given by

(6) $$\begin{array}{lll} \;b_{i,k}|s,s & \neq 1,\mu_{b,g,k},\sigma_{b,g,k}\; & \;\\ & ∼(1-\varepsilon_{b,k})\underset{g\in GT_{s}}{\sum }p_{i,g}\left\{TN(b_{i,k}|\mu_{b,k,g},\sigma_{b,k,g})\right\}\;\\ & \;+\varepsilon_{b,k}\;U(b_{i,k}|0,1)\;,\; & \; \end{array}$$

where the truncated-normal ( TN) mixture captures within-sample heterogeneity of the B allele frequencies over the possible genotypes for state *s* (*GT*_*s*_) and the uniform zero-one density captures technical variation that we assume to be independent of the genotype and copy number state. As B allele frequencies are thresholded to the [0,1] interval, the proportion of outlier log R ratios, *ε*_*r*,*k*_, does not necessarily correspond to the proportion of outlier B allele frequencies given by *ε*_*b*,*k*_, motivating their separate parameterization. The mixture probability *p*_*i*,*g*_ is estimated from a binomial density parameterized by the frequency of the A allele for genotype *g* (i.e., 2 for genotype *AA*) and the population frequency of the A allele. Estimation of the parameters for the means, variances, and outlier mixture probabilities for the B allele frequencies are estimated via the Baum-Welch algorithm as described in the Methods section. For the homozygous null state, we assume the B allele frequencies are emitted from a uniform zero-one distribution.

The likelihood in equations (3) and (4) is multiplied by terms involving the conditional probability of the offspring copy number, the initial state probability of the parental copy numbers (if *l *= 1), and transition probabilities for the parental copy numbers (if *l *> 1). We calculate the conditional probability for the offspring copy number by integrating out (averaging over) Mendelian and non-Mendelian models for CNV transmission. The derivation of the conditional probability is similar to the derivation in the joint HMM, but indexed over segments instead of markers. We leave the mathematical details to Additional file 1 (see also [40]) and specification of the initial state and transition probabilities to Section Methods. Multiplication of these terms with the likelihood provides an estimate of the posterior probability. Repeating the estimation procedure for each of the 121 possible trio states, we obtain a distribution of the posterior probability. The mode of this distribution is the maximum a posteriori estimate. Conditional on the maximum a posteriori estimate at segment *l*, we repeat the procedure for segment *l* + 1 until maximum a posteriori estimates are available for all segments.

Segmentation and maximum a posteriori estimation are performed independently for each chromosomal arm and each trio, enabling an embarrassingly parallel implementation. Computational speed is derived from the parallel architecture and the implementation of the computationally intensive maximum a posteriori estimation (121 calculations) on a set of segments that is typically several orders of magnitude smaller than the number of markers on the array.

### Simulation study

To assess the performance of PennCNV and MinimumDistance when the true CNV are known, we simulated chromosomes containing four de novo and four inherited copy number deletions spanning as few as 10 markers and as many as 100 markers. We additionally simulated three regions of homozygosity of 50, 100, and 500 markers in the offspring that were diploid in copy number and spanned by the trough of a simulated wave (see Methods). Log R ratios for a trio were sampled from a 3-dimensional multivariate normal distribution under 12 different parameterizations of the covariance for the trio (see Methods). B allele frequencies for the offspring were simulated to be consistent with Mendelian transmission.

**Figure 2:**
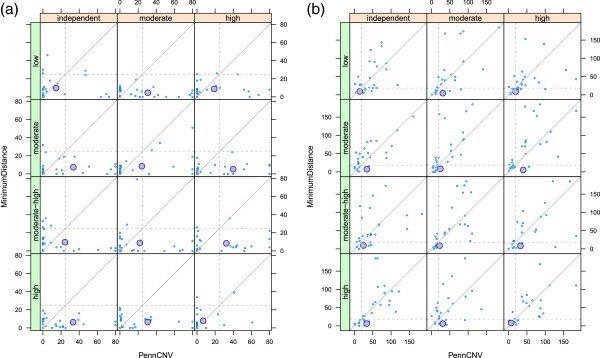
**Performance of PennCNV and MinimumDistance on simulated data.** Each point represents a synthetic 25,000 basepair chromosome in which the number of markers incorrectly called de novo **(a)** or not de novo **(b)** were tabulated for PennCNV and MinimumDistance. Log R ratios were simulated with three different levels of correlation between individuals in the trio (columns) and four different levels of variance (rows). The diagonal line in each panel is the identity. **(a)** False positive frequencies in PennCNV and MinimumDistance are uncorrelated, with more skewed frequencies in PennCNV that were threshold at 80 to fit on the display. The mean false positive frequency in MinimumDistance is lower than PennCNV over a range of variance and correlation settings (large circles). The gray horizontal and vertical dashed lines correspond to false positive rates of 0.001. **(b)** The number of markers falsely called de novo is highly correlated between methods. The mean false negative frequency is comparable in PennCNV and MinimumDistance (large circles). The gray horizontal and vertical dashed lines denote false negative rates of 0.1.

We define false positives (FP) as the number of markers in normal regions called de novo and false negatives (FN) as the number of markers in de novo regions called normal. Overall, the correlation of FP for MinimumDistance and PennCNV was low. On average, the FP frequency is higher for the joint HMM than for MinimumDistance with several chromosomes having relatively high FP in PennCNV and low FP in MinimumDistance (bottom right quadrants of panels in Figure 2a). For synthetic chromosomes in which both methods have low FP rates, PennCNV does slightly better than Minimum Distance (bottom left quadrants for panels in Figure 2a). The low FP rate for at least one method in nearly all of the simulations suggests that de novo CNVs called by both methods on experimental data are more likely to be truly de novo. Unlike FP frequencies, FN frequencies for PennCNV and MinimumDistance are comparable with both methods doing well (lower left quadrants) or poorly (upper right quadrants) on the same chromosome (Figure 2b).

To assess how incorrect calls were distributed among the different CNVs, we calculated the proportion of the 25 chromosomes for which 50 percent or more of the markers in the CNV were classified incorrectly. None of the transmitted deletions had more than 50% of the markers called de novo by either method. Diploid regions of homozygosity had elevated FP rates in PennCNV, although the difference was not statistically significant (data not shown). For de novo CNV, MinimumDistance correctly called a higher percentage of the 10-marker features than PennCNV (column 1, Additional file 2: Figure S2), while PennCNV performed well relative to MinimumDistance for detecting large de novo features under simulations with high log R ratio variance (bottom right panel, Additional file 2: Figure S2). Approximately 80 percent of the oral cleft trios had MADs less than 0.2, a scenario in which MinimumDistance FN rate was comparable or better than PennCNV (rows 1 and 2, Additional file 2: Figure S2).

### Case study of oral clefts

We assessed the performance of MinimumDistance and PennCNV on a set of oral cleft trios obtained from the International Consortium of Oral Clefts and genotyped on Illumina’s 610 quad array as part of the Gene, Environment, Association Studies consortium [46]. From a computational vantage point, MinimumDistance was clearly preferable. PennCNV’s joint HMM required an average of 130 minutes for a single trio. Without parallel processing, the MinimumDistance algorithm required an average of 17 minutes to process 22 autosomes of a single trio and approximately 3 minutes using 22 CPUs. One trade-off is that MinimumDistance uses approximately 17G RAM while PennCNV requires less than 3G RAM. In practice, the increase in computational speed using MinimumDistance will depend on I/O, the number of CPUs available, and RAM constraints.

**Figure 3:**
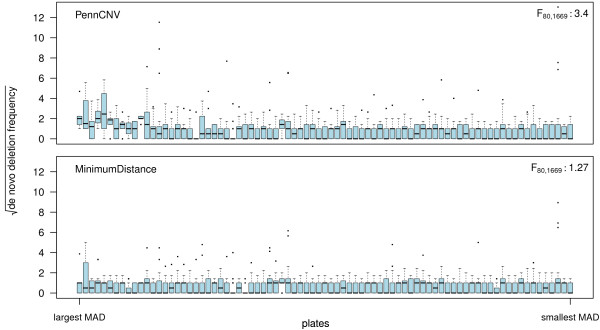
**Plate-effect for de novo deletion frequencies.** The square root of de novo deletion frequencies stratified by chemistry plate for PennCNV (top) and MinimumDistance (bottom). Plates are ordered by the median MAD from high (left) to low (right). F-statistics from an analysis of variance of the square root frequencies by plate are displayed in top right legend of each panel.

When assessing the concordance of de novo hemizygous deletions called by MinimumDistance and PennCNV on 1,741 oral cleft trios that passed quality control (see Methods), we found that the 50^th^ and 75^th^.. the 95^th^ and 99^th^ corresponding to 5 and 23 de novo alterations, respectively, in PennCNV compared to 2 and 7.5 alterations in MinimumDistance. MinimumDistance called a total of 1,261 de novo deletions in 651 trios versus 3,006 de novo deletions in 824 trios called by PennCNV. Nearly 40 percent of the PennCNV de novo deletions (1,174) occur in just 12 percent (212) of the trios. The 212 trios that harbor 40 percent of the de novo deletions were processed on the 15 chemistry plates having the highest log R ratio MAD (top, Figure 3). The Spearman correlation coefficient of the plate-wise mean MAD and the plate-wise mean de novo frequency is 0.54, suggesting a batch effect induced by differences in noise across plates even after quality control. Conversely, MinimumDistance calls 96 de novo deletions (8 percent of the total calls) in the same trios (bottom, Figure 3).

**Figure 4:**
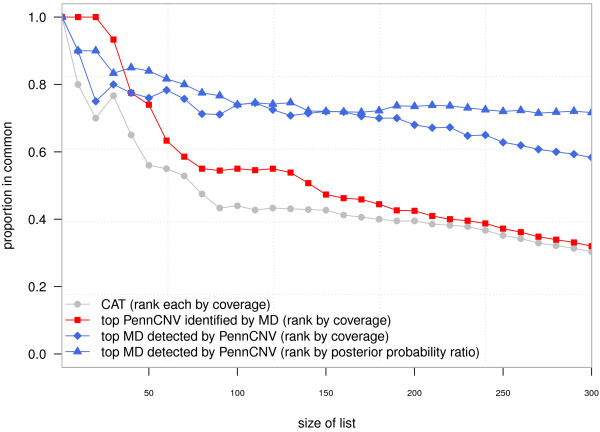
**Concordance of PennCNV and MinimumDistance as a function of list size.** De novo hemizygous deletions identified by PennCNV and MinimumDistance were ranked by coverage. Plotted on the vertical axis is the proportion of de novo hemizygous deletions identified by both methods as a function of list size. For concordance at the top (CAT), the proportion in common is calculated for the top hits in each list (gray circles). We also plot the proportion of top hits detected by one method that were called de novo by the second method (squares and diamonds). Ranking the MinimumDistance list by the ratio of the maximum a posteriori probability to the posterior probability of diploid copy number improved the concordance (≈ 75%).

To systematically evaluate concordance of PennCNV and MinimumDistance, we created a list of the de novo deletions for each method ordered by decreasing coverage. We assessed concordance using three complimentary approaches: (i) the concordance at the top (CAT) defined as the proportion of de novo deletions appearing in the top of both lists [47], (ii) the proportion of top PennCNV de novo deletions appearing anywhere in the MinimumDistance list, and (iii) the proportion of top MinimumDistance de novo deletions appearing anywhere in the PennCNV list. Plotting the concordance as a function of list size, the CAT decreases from 100% for a size one list to 44% for a size 100 list (gray circles, Figure 4). Whereas 77 of the top 100 MinimumDistance hits are corroborated by PennCNV (blue diamonds), only 53 of the top 100 PennCNV hits are corroborated by MinimumDistance (red squares). While the proportion of top MinimumDistance calls detected by PennCNV is 60 percent for a list of size 300 and trending downward, the concordance stabilizes at 76 percent when MinimumDistance calls are ranked by the ratio of the maximum a posteriori probability to the posterior probability of diploid copy numbers in the trio (blue triangles).

For de novo deletions with high coverage called by only one method, many appear to be artifacts with the number of apparent false positives in PennCNV nearly double that of MinimumDistance. As in the motivating example (Figure 1), PennCNV-only de novo deletions tend to have troughs that are shared by members in the trio (see Additional file 2: Figures S3–S8). Apparent false positives in the MinimumDistance-only calls occur in complex CNV in which the minimum distance breakpoints may span both de novo and transmitted CNV or as a result of genomic waves that are slightly inverted in the offspring compared to the parents (see S9-S13). MinimumDistance captures at least one region in which the de novo CNV appears to be a false negative in PennCNV (Additional file 2: Figure S14).

**Figure 5:**
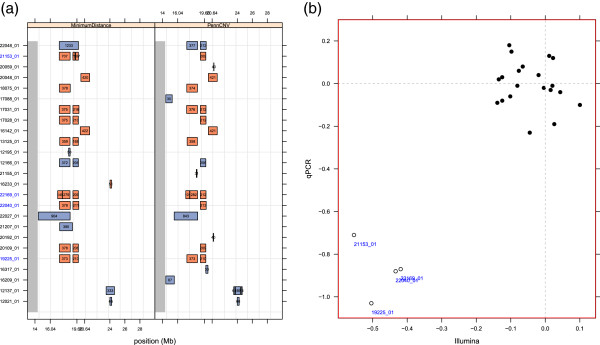
**Concordance assessment of methods (a) and platforms (b) for de novo CNVs in the DiGeorge critical region on chromosome 22. (a)** The physical position of de novo deletions and amplifications for 25 trios are indicated by orange and blue boxes, respectively, for MinimumDistance (left panel) and PennCNV (right panel). Boxed numbers indicate the number of Illumina markers. Blue y-axis labels indicate trios validated by qPCR. **(b)** Each point is the minimum distance of the CopyCaller^TM^ copy number estimates (y-axis) from the qPCR platform and the minimum distance of the spanning CBS segment from the Illumina platform (x-axis) for one trio. A total of eight trios and three TaqMan probes were used in the validation experiment, generating 24 points on the scatterplot. The inter-platform concordance is high as indicated by the clusters at the bottom left and top right of the display. The four trios at the bottom left have a putative de novo CNV called by MinimumDistance and PennCNV at the 19.60 Mb locus. TaqMan probes flanking the de novo deletion (16.04 and 20.64 Mb) for these trios have minimum distance estimates that cluster near zero (top right). The top right cluster also contains four trios for whom the putative copy numbers were inferred to be diploid by MinimumDistance and PennCNV at the 19.60 Mb locus.

In terms of concordance, de novo CNVs identified by both methods appear to be more amenable to experimental validation. Nearly half of the 40 concordant de novo calls that rank high by each method in terms of coverage occur on chromosome 22 (Figure 5a). Visual inspection of the log R ratios, B allele frequencies, and minimum distance for these trios is consistent with the de novo inference (e.g., Additional file 2: Figure S15). Using qPCR for experimental validation of the apparent de novo CNVs in four trios, we summarize the inter-platform concordance by the minimum distance of the log R ratio segment means from the Illumina platform and the minimum distance of the CopyCaller^TM^ copy number estimates from the qPCR platform [48]. A scatterplot of the minimum distance reveals two clusters separating in both the x- and y-dimensions according to whether the probes on the Illumina (y-axis) and the Taqman qPCR probes (x-axis) are spanned by the de novo CNV (bottom left) or flank the de novo CNV (top right, Figure 5b). This region on chromosome 22, known as the DiGeorge critical region, has been previously implicated in syndromic forms of cleft palate [49-52].

## Conclusions

Genomic wave correction in conjunction with the joint HMM for case-parent trios is perhaps the de facto analysis for inferring de novo CNV, yet we find a number of de novo calls that appear to be artifacts of genomic waves and call rates that are correlated with batch (chemistry plate). We propose a simple, univariate measure of relative copy number that can reduce local and global sources of heterogeneity such as probe-effects and genomic waves, respectively, and can be segmented by standard, single-sample segmentation algorithms. We use the method of maximum a posteriori estimation for inferring the de novo status of segments. Key terms in the posterior probability are the likelihood, which we estimate robustly, and the probability of the offspring copy number conditional on the parental copy numbers. We compute the latter term by integrating over Mendelian and non-Mendelian models for CNV transmission, using tabled probabilities from the joint HMM directly for the Mendelian model. The MinimumDistance algorithm is several-fold faster than the joint HMM without any apparent trade-off in sensitivity or specificity as assessed by simulation. Unlike PennCNV, the frequency of de novo calls by MinimumDistance appears robust to differences in noise across batches and robust to genomic waves occurring in trios. De novo calls with high coverage that were concordant between methods include several de novo deletions and amplifications in the DiGeorge critical region on chromosome 22, four of which were subsequently validated by qPCR. As the DiGeorge critical region is known to be important for syndromic disorders that include craniofacial abnormalities, the de novo deletions from independent trios with non-syndromic oral cleft may help identify genes responsible for oral clefts. This finding, verifiable by both de novo detection algorithms, was obtained with a nearly 8-fold reduction in computational time using MinimumDistance.

Our approach for de novo CNV detection can have several limitations. First, the set of candidate breakpoints identified by segmenting the minimum distance are relevant only for identifying genomic regions in which the offspring copy numbers differ from the parental copy numbers. Breakpoints for transmitted CNV are only detectable when the copy number estimates within the CNV differ in magnitude between parents and offspring. Secondly, while genomic waves are strongly correlated with GC content, differences in direction or magnitude of waves across samples are not uncommon. Previous studies suggest that differences in DNA quantity contribute to inversions of the genomic waves between samples [7]. While we observed that the waves were often comparable within a trio, this assumption requires verification. To the extent that we can detect inversions, future versions of MinimumDistance may provide warnings of such artifacts or apply methods to correct for those artifacts. Finally, the MinimumDistance algorithm is only defined for autosomal chromosomes.

A potential criticism of the current study is that we have evaluated a novel method on a dataset that has not been well studied for CNVs in the literature. While HapMap has been comprehensively characterized by several platforms and statistical methods, there are limitations. First, the cell lines used in HapMap studies have a signal to noise ratio much higher than the signal to noise ratio observed in DNA isolated from experimental studies such as the oral cleft dataset. In fact, our approach was motivated by the technical variation shared among trios in the oral cleft study. Secondly, a recent study failed to identify de novo CNVs in HapMap, identifying instead somatic changes or possible problems with the cell lines [38]. Finally, while one could conceptually use the available HapMap trios to derive a null distribution for the frequency of recurrent de novo CNV in healthy populations, the practical benefit of such a null would be limited as many of the recurrent de novo regions in the oral cleft study occur in fewer than 1 in 100 offspring. Due to these limitations and the absence of confirmed de novo CNVs in both the oral cleft study and HapMap, we have evaluated the methods by simulation and visual inspection of the low-level summaries. One consequence of the latter is that we avoid low coverage de novo calls as visual inspection of such regions tend to be inconclusive.

## Methods

### Case study samples and data

The case-parent trio study for oral clefts is part of the Gene, Environment Association Studies consortium, commonly known as GENEVA [46,53]. High-throughput genotyping was performed at the Center for Inherited Disease Research using Illumina’s 610 quad array. Raw intensities from the scanned arrays were preprocessed and summarized using BeadStudio software version 3.3.7 as described previously [53]. The joint HMM was implemented in PennCNV (version May, 2010) and copy number estimates from qPCR was obtained using CopyCaller^TM^ (v2.0). All other statistical analyses were performed using the statistical environment R[54]. The version of R and various R packages used in our analysis are indicated in Additional file 1. Genomic annotation is based on build hg18 of the UCSC Genome Browser database [55].

### Quality control

We applied the joint HMM implemented in PennCNV with wave correction to 6,202 samples comprising 2,082 nuclear families in the oral cleft study. Using default settings for PennCNV, 560 samples were flagged for log R ratio standard deviations exceeding 0.3, B allele frequency drift greater than 0.01, or wave factor greater than 0.05 [7]. Of the flagged samples, approximately 20% were whole genome amplified at the collection site. Whole genome amplification suggests insufficient DNA and de novo call frequencies were elevated 50-fold in these trios relative to non-whole genome amplified samples (Additional file 2: Figure S16). We excluded 341 trios in which one or more samples had whole genome amplified DNA, a log R ratio MAD greater than 0.3, or flagged by either of the PennCNV statistics for drift and waves. While trios for which the DNA source was not whole blood have higher log R ratio MAD and higher de novo call rates (Additional file 2: Figure S16), only whole genome amplified DNA source was explicitly excluded. For the 5,216 samples passing quality control, 92 percent (4,826 samples) had DNA derived from whole blood.

### MinimumDistance

The minimum distance was computed directly from BeadStudio log R ratios. We applied CBS independently to each chromosomal arm using default values of the segment function in the R package DNAcopy [12]. To promote breakpoints flanking gaps in coverage, we implement CBS independently to chromosomal regions that have an inter-marker distance of less than 75,000 basepairs. If a chromosomal region contained fewer than 1000 markers, the gap was ignored and the region may include markers separated by more than 75,000 basepairs. For example, CBS was fit independently to 14 regions of chromosome 1. A similar binning strategy was used for lowess smoothing of ratios of log intensities to estimate copy number in a spike-in experiment [56]. Applying CBS independently to regions flanking gaps in coverage has a small computational cost as the number of candidate de novo segments will be more than the number of segments identified without splitting across gaps in coverage. Users of the software can choose an alternative distance, or select an arbitrarily large distance such that the segmentation is run on the entire chromosomal arm.

Estimation of the likelihood of the resulting segments requires parameterizing the mixture distributions for the log R ratios and B allele frequencies (see equations 5 and 6, Section Results and discussion). Initial versions of MinimumDistance used theoretical means shared by all samples and estimated the log R ratio variances using an empirical Bayes approach that incorporated a term for the cross-sample variance at each marker. Disadvantages of this approach included means that were less robust to departures from the theoretical values and inflated variance estimates for copy number polymorphic regions due to the higher variability of the log R ratios across samples. These observations led us implement the Baum-Welch algorithm to update parameters *μ*_*b*,*k*,*g*_, *σ*_*b*,*k*,*g*_, *ε*_*b*,*k*_, *μ*_*r*,*k*,*s*_, *σ*_*r*,*k*,*s*_, and *ε*_*r*,*k*_ from their initial values (see equations (6) and (5)). Issues of identifiability and our desire to parallelize across chromosomes for computational speed have led to several constraints for the Baum-Welch update (see Additional file 1 for initial values and constraints).

To calculate posterior probabilities, the likelihood is multiplied by the initial state probability of the parental copy numbers (if *l*=1), the transition probabilities for the parental copy numbers (for segments *l*>1), and a conditional probability for the offspring copy number. We assumed that any of the 5 copy number states were equally probable for the initial state probability. For the transition probability, we use $\frac{1}{2}$ when the states of adjacent segments are the same and $\frac{1}{8}$ otherwise. To calculate the conditional probability for the offspring, we integrate over a latent, binary indicator for Mendelian transmission. Our approach is similar to the factorization in the joint HMM [40], but over segments instead of markers. To illustrate, we derive the joint probability of the trio copy number state ***s***_1_ for the first segment, P(***s***_1_|**Θ**), in Additional file 1. Integrating the conditional probability of the offspring copy number over Mendelian and non-Mendelian models requires (i) an estimate of the probability of the offspring copy number conditional on the parental copy numbers under the Mendelian model, (ii) an estimate of the marginal probability of the offspring copy number under the non-Mendelian model (the offspring copy number is independent of the parental copy numbers), and (iii) the probability of the Mendelian model. For (i), we use previously published tabled probabilities (Additional file 1: Table 1, [40]). For (ii), we assume that any of the copy number states are equally probable. For (iii), we use 1−1*.*5×10^−6 ^as in the joint HMM. Details regarding the conditional joint probability for segments *l *> 1 are included in Additional file 1.

### Simulation

We simulated chromosomes of 25,000 markers containing four de novo and four inherited copy number deletions that differ in the number of markers: 10, 25, 50, or 100 markers. In addition, we simulated three regions for which the offspring genotypes were homozygous with copy number two. Coverage in the three regions of homozygosity was 50, 100, and 500. Parameters of our simulation are the means and covariance of a three-dimensional multivariate normal distribution from which the log R ratios for a trio were sampled. Off-diagonal elements of the 3×3 correlation matrix of the trio were assumed to be the same with settings corresponding to independence (*ρ *= 0), moderate correlation (*ρ *= 0*.*2), and high correlation (*ρ *= 0*.*5). For each correlation, four levels of standard deviation were simulated: low (*σ*_*r *_= 0*.*15), moderate (*σ*_*r *_= 0*.*20), moderate-high (*σ*_*r *_= 0*.*25), and high (*σ*_*r *_= 0*.*30). The standard deviation and correlation parameters were selected based on the corresponding empirical estimates of these parameters in the oral cleft study (see Additional file 1).

For de novo hemizygous deletions, the mean for the parental log R ratios is zero and the mean for the offspring log R ratios is -0.5, approximating what we observe empirically. For transmitted deletions, the log R ratios for the father and offspring were simulated from normal distributions with mean -0.5. To simulate genomic waves spanning regions of homozygosity, we changed the mean smoothly as a function of the marker index along the chromosome from 0.0 to -0.2 to simulate a smooth wave. The correlation parameter of the log R ratios for each father-mother-offspring pair is the same. For deletions and genomic wave features, the B allele frequencies were simulated to be consistent with Mendelian inheritance of the transmitted allele(s). Twenty-five synthetic chromosomes were simulated for each covariance matrix.

## Abbreviations

SNP: Single nucleotide polymorphism; aCGH: Array comparative genomic hybridization; HMM: Hidden Markov model; MCMC: Markov chain Monte Carlo; CNV: Copy number variant; CBS: Circular binary segmentation; FP: False positive; FN: False negative MAD: Median absolute deviation (MAD); qPCR: Quantitative polymerase chain reaction; CAT: Concordance at the top; GENEVA: Gene-Environment Association Studies consortium.

## Competing interests

The authors declare that they have no competing interests.

## Authors’ contributions

RBS, IR, AFS, SGY, HS, and THB conceived of the study and participated in the drafting of the manuscript. RBS performed the data analysis and wrote the software. HS carried out the analysis of the PennCNV algorithm and provided useful suggestions for the manuscript. AFS performed qPCR validation of candidate de novo regions. SGY contributed to the development of the MinimumDistance R package. All authors read and approved the final manuscript.

## Supplementary Material

Additional file 1 **Optional post-processing of CBS segments.** Removing splits from CBS as a function of coverage and the standardized difference in segment means. **Baum-welch updates** Initialization and updating of parameters for the emission distributions. **Models for Mendelian transmission of the offspring copy number** Details regarding the adaption of the PennCNV probabilistic model of Mendelian transmission. **PennCNV annotation for trio copy number states** Annotation of trio copy number states in PennCNV. **Empirical estimation of simulation parameters in the oral cleft study** Estimation of simulation parameters from the oral cleft study **Renvironment and software versions** Click here for file

Additional file 2 **Supplementary figures.** Supporting figures. Click here for file
