# Supplementary material for: Fast detection of de novo copy number variants from SNP arrays for case-parent trios
Source: BMC Bioinformatics. 2012 Dec 12;13:330. doi: 10.1186/1471-2105-13-330 (PMC3576329; doi:10.1186/1471-2105-13-330)
Supplement: Additional file 1 — Optional post-processing of CBS segments. Removing splits from CBS as a function of coverage and the standardized difference in segment means. Baum-welch updates Initialization and updating of parameters for the emission distributions. Models for Mendelian transmission of the offspring copy number Details regarding the adaption of the PennCNV probabilistic model of Mendelian transmission. PennCNV annotation for trio copy number states Annotation of trio copy number states in PennCNV. Empirical estimation of simulation parameters in the oral cleft study Estimation of simulation parameters from the oral cleft study Renvironment and software versions [file 1471-2105-13-330-S1.pdf]

Contents

Optional post-processing of CBS segments . . . 1
Baum-Welch updates . . . 1
Models for Mendelian transmission of the offspring copy number . . . 5
PennCNV annotation for trio copy number states . . . 7
Empirical estimation of simulation parameters in the oral cleft study . . . 7
R environment and software versions . . . 8

Optional post-processing of CBS segments

Following segmentation, CBS provides a post hoc option to remove splits when the standardized difference in adjacent segment means is less than a user-specified cutoff. A related approach is to define a rule for removing splits such that the cutoff is a function of the standardized difference in segment means as well as the number of markers on a segment (referred to as coverage). Scaling the difference in segment means by the median absolute deviation (MAD) of the autosomal log R ratios, the dependency of the cutoff on the MAD and coverage can be expressed by a simple exponential rule, such as the exponential curve in Figure 1. For each pair of adjacent segments, coverage of the $5'$ segment and the scaled difference in the segment means defines an x-y coordinate in the scatterplot. For segment pairs below the exponential curve, the two segments are combined into a single segment. The procedure is applied recursively to each chromosome. Currently, the implementation to remove splits is optional in MinimumDistance as we have found combining segments that have the same trio copy number classification often reduces the need for this step.

Baum-Welch updates

An overview of the MinimumDistance pipeline for calling de novoCNVs is provided in Figure 2. This section describes the procedure for updating model parameters for maximum a posteriori estimation of the trio copy number states.

We have posited normal-uniform mixture distributions for the log R ratios and B allele frequencies (equations (5) and (6), Section Results and discussion). The parameters for the mixture distributions are sample-specific, as the mean, variances, and outlier probabilities in the mixture often vary from sample to sample as a result of differences in the quality and quantity of isolated DNA as well as other latent factors (e.g., lot numbers of experimental reagents). We specify initial values for these parameters that are common to all samples, but update these parameters using the iterative Baum-Welch algorithm [1]. Initial values

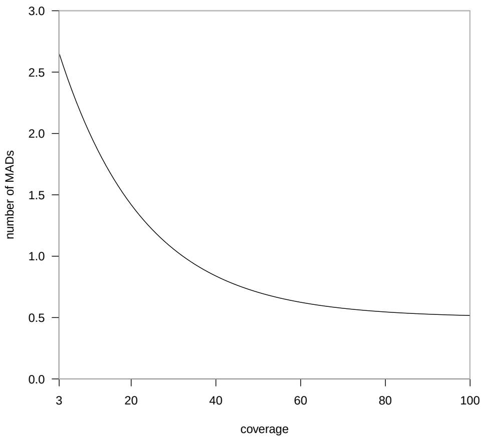

Figure 1: For each pair of adjacent segments on a chromosome, we calculated (i) the difference in the segment means standardized by the median absolute deviation of the minimum distance across all autosomal markers and (ii) the coverage of the $5'$ segment of the pair. Coverage and the standardized difference in means define an x,y coordinate in the above plots. A simple exponential curve, here $0.05 + 0.05e^{-0.05\times\text{coverage}}/0.02$, can be used to express the dependency of the cutoff on coverage. For pairs of adjacent segments falling below the exponential curve, the split is removed and a new segment is created by combining the adjacent segments. For each chromosome, the procedure is applied recursively until no splits are removed.

for the means and variances in our analysis of the oral cleft data are provided in Tables 1a and 1b; outlier probabilities were initialized to 0.01 for log R ratios and 10^{-5} for B allele frequencies. For computational speed, we parallelize the Baum-Welch update across chromosomal arms. Hence, $\mu_{b,g}$, $\sigma_{b,g}$, ϵ_b, $\mu_{r,s}$, $\sigma_{r,s}$, and ϵ_r are updated independently for each chromosomal arm and each sample. To avoid overfitting and to ensure identifiability, we impose several constraints that we describe in detail below. We emphasize that the Baum-Welch update is performed to robustly estimate parameters for the mixture distributions used in the calculation of the likelihood.

An implementation of the Baum-Welch algorithm for genotyping arrays is available in the R package VanillaICE available from bioconductor [2, 3]. Transition probabilities for the Baum-Welch update are specified as a function of the distance between markers (see [2]). Initial state probabilities are assumed to be the same for all states. Neither the transition probabilities nor the initial state probabilities are updated by the Baum-Welch algorithm in this implementation. Forward (α) and backward (β) probabilities are efficiently computed using the Viterbi algorithm [4].

State-specific weights for the sequence of log R ratios are functions of α and β. Specifically, the weight

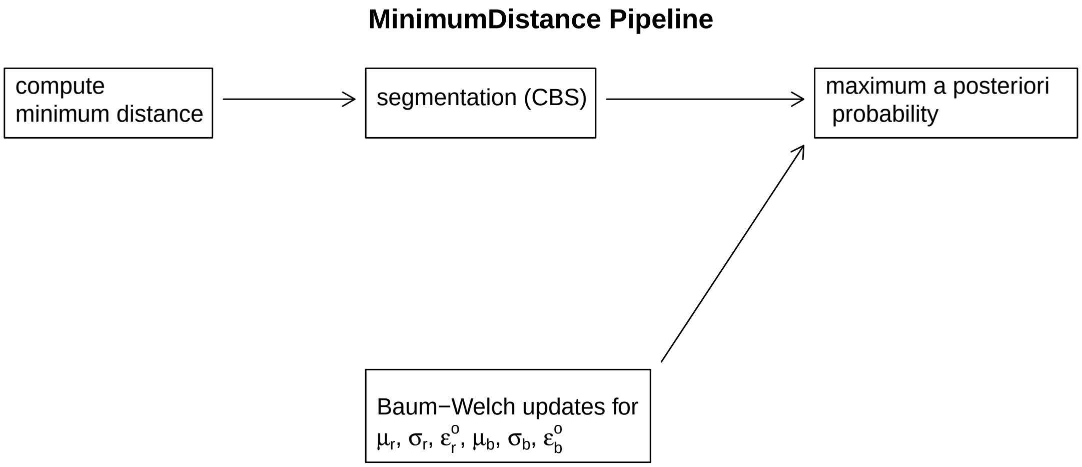

Figure 2: Components of the MinimumDistance pipeline for de novo CNV inference.

is the probability that the log R ratio r at marker i is emitted from state s and is given by

$$w_{r,i}(s) = \frac{\alpha_i(s)\beta_i(s)}{\sum_i \alpha_i(s)\beta_i(s)} N(r_i|\mu_{r,s}, \sigma_{r,s}). \tag{1}$$

For clarity, the above weights are for a single sample and we have omitted the sample index in the notation. The updated means and standard deviations are given by

$$\bar{\mu}_{r,s} = \frac{\sum_{i=1}^M w_{r,i}(s) r_i}{\sum_{i=1}^M w_i(s)} \quad \text{and} \quad \bar{\sigma}_{r,s} = \sqrt{\frac{\sum_{i=1}^M w_{r,i}(s)(r_i - \mu_{r,s})^2}{\sum_{i=1}^M w_{r,i}(s)}},$$

respectively.

For the B allele frequencies, the probability that genotype g of state s at marker i accounts for the emitted observation is given by

$$w_{b,i}(s,g) = \frac{\alpha_i(s)\beta_i(s)}{\sum_{i=1}^M \alpha_i(s)\beta_i(s)} \frac{p_{i,g}\mathcal{TN}(b_i|\mu_{b,g}\sigma_{b,g})}{\sum_{g \in G_s} p_{i,g}\mathcal{TN}(b_i|\mu_{b,g}\sigma_{b,g})},$$

where $\mathcal{TN}$ denotes the truncated normal distribution (truncated to the interval [0,1]). The reestimation formulas for the B allele frequency mean and standard deviation for genotype g are given by

$$\bar{\mu}_{b,g} = \frac{\sum_{i=1}^M w_{b,s}(g) b_i}{\sum_{i=1}^M w_{b,s}(g)} \quad \text{and} \quad \bar{\sigma}_{b,g} = \sqrt{\frac{\sum_{i=1}^M w_{b,s}(b_j - \mu_{b,g})^2}{\sum_{i=1}^M w_{b,s}}},$$

respectively.

Several constraints are required to ensure identifiability and to avoid overfitting. Minimum (l_r) and maximum (u_r) values of the uniform outlier-component of the mixture distribution for the log R ratios are

set to -2.5 and 3.0, respectively [see equation (5), Section Results and discussion]. Log R ratios exceeding the upper and lower bounds of the uniform are thresholded. While a truncated normal distribution may be more appropriate for estimating the density, in practice we routinely remove samples with high log R ratio variance as part of quality control. We assume that the standard deviations for all positive copy number states is the same. To estimate the standard deviation for positive copy number states, we first compute the copy number state with the largest cumulative weight,

$$s' = \underset{s \in \{1,2,3,4,5,6\}}{\arg\max} \sum_i w_{r,i}(s).$$

As many log R ratios are likely to have been emitted from state s', we assume that the standard deviation of positive copy number states can be well estimated by $\bar{\sigma}_{r,s'}$. The outlier probability for the log R ratios is estimated as the mean number of observations for which $w_{r,i}(s) < c_r$ for all states s with the constraint that $\bar{\epsilon}_r^o \leq 0.1$. The constraint ensures for ϵ_r ensures that the outlier component does not absorb too much of the variance. We set $c_r = 0.1$.

For B allele frequencies, we fix the mean for homozygous A and homozygous B genotypes at 0 and 1, respectively, and we do not update these values (i.e., $\mu_A = \mu_{AA} = \mu_{AAA} = \mu_{AAAA} = 0$). The means for heterozygous genotypes are updated but constrained such that the means are non-decreasing for genotypes with increasing proportions of B alleles ($\mu_A < \mu_{AAAB} < \mu_{AAB} < \mu_{AB} < \mu_{ABB} < \mu_{ABBB} < \mu_B$). The B allele frequency means for heterozygous genotypes AB and AABB are constrained to the interval [0.45, 0.55]. For the set of heterozygous genotypes denoted by HET ($HET \in$ {AB, AAB, AAAB, AABB, ABBB}), we find the genotype with the largest cumulative weight across all markers as

$$g' = \underset{g \in HET}{\arg\max} \sum_{s \geq 3} \sum_i w_{r,i}(s, g).$$

We update the B allele frequency standard deviations for all heterozygous genotypes as $\bar{\sigma}_{b,g'}$. The probability of an outlier B allele frequency is updated as

$$\bar{\epsilon}_b = \frac{1}{M} \sum_i \mathbb{I}_{[w_{b,i}(s) < c_b]} ,$$

where M is the number of markers. We set $c_b = 0.9$ and constrain $\bar{\epsilon}_r^b \leq 0.01$.

We iteratively implement the Baum-Welch algorithm until the difference in the likelihood of the observed data between successive iterations of the Viterbi algorithm is suitably small, or a maximum number of updates has been reached. Typically, three or four iterations are sufficient for the estimates to stabilize. The likelihood of the observation sequences for the log R ratios and B allele frequencies for the lth minimum

State (s)	$\mu_{r,s}$	$\sigma_{r,s}$
homozygous deletion	-2.0	3ω
hemizygous deletion	-0.4	ω
diploid	0.0	ω
diploid, region of homozygosity	0.0	ω
single copy gain	0.4	ω
two+ copy gain	1.0	ω

(a)

Genotype (g)	$\mu_{b,g}$	$\sigma_{b,g}$
homozygous A	$0.0^{\dagger}$	0.02
homozygous B	$1.0^{\dagger}$	0.02
AAAB	0.25	0.05
AAB	0.33	0.05
AB or AABB	0.50	0.05
ABB	0.67	0.05
ABBB	0.75	0.05

(b)

Table 1: (a) Initial values for the mean and standard deviation of the log R ratios. The standard deviation, ω, is estimated from the data as the MAD of the log R ratios. (b) Initial values for the mean and standard deviation of the B allele frequencies. Homozygous genotypes for the same allele (e.g., A, AA, AAA, and AAA) are assumed to have the same mean and standard deviation. Baum-Welch updates for $\mu_{b,g}$ are constrained to be strictly increasing with the proportion of B alleles (e.g., $\mu_{b,AAB} < \mu_{b,AB} < \mu_{b,ABB} < \ldots$). We constrain $\sigma_{b,g}$ to be the same for all heterozygous genotypes (rows 3 - 7). †not updated as part of the Baum-Welch algorithm.

distance segment for each state is calculated as a product over markers on the segment and samples in the trio. Finally, the Baum-welch update implemented in the R package VanillaICE includes a sixth state for diploid regions of homozygosity. We calculate the likelihood of diploid copy number state as

$$\max\left\{\mathsf{P}(b_l, r_l|s_k = 3, \mathbf{\Theta}), \mathsf{P}(b_l, r_l|s_k = 4, \mathbf{\Theta})\right\},$$

where $s = 3$ is the state for diploid copy number and normal heterozygosity and state $s = 4$ corresponds to diploid copy number for a region in which all (or nearly all) of the genotypes are homozygous.

Models for Mendelian transmission of the offspring copy number

For the first segment, the likelihood is multiplied by the probability of the trio copy number state, $\mathsf{P}(\boldsymbol{s}_1|\mathbf{\Theta})$. This probability can be factored as a product of the initial state probability of the parental copy numbers and a conditional probability for the offspring copy number. We assume that any of the 5 copy number states are equally probable for the initial state probabilities. For the offspring copy number, we integrate the

conditional probability over Mendelian and non-Mendelian models for transmission by introducing a latent, binary indicator for non-Mendelian copy number denoted as NM_l. This is comparable to the likelihood in the joint HMM [5], but over segments instead of markers. Taken together, we write

$$\begin{aligned}
\mathsf{P}(\boldsymbol{s}_1|\boldsymbol{\Theta}) &= \sum_{\text{NM}_1=0}^{1} \mathsf{P}(\boldsymbol{s}_1, \text{NM}_1|\boldsymbol{\Theta}) \\
&= \sum_{\text{NM}_1=0}^{1} \mathsf{P}(\boldsymbol{s}_1|\boldsymbol{\Theta}, \text{NM}_1)\mathsf{P}(\text{NM}_1|\boldsymbol{\Theta}) \\
&= \sum_{\text{NM}_1=0}^{1} \mathsf{P}(s_{1,O}|s_{1,F}, s_{1,M}, \text{NM}_1, \boldsymbol{\Theta})\mathsf{P}(s_{1,F}|\boldsymbol{\Theta})\mathsf{P}(s_{1,M}|\boldsymbol{\Theta})\mathsf{P}(\text{NM}_1|\boldsymbol{\Theta}) \\
&= \left(\frac{1}{5}\right)^2 \sum_{\text{NM}_1=0}^{1} \mathsf{P}(s_{1,O}|s_{1,F}, s_{1,M}, \text{NM}_1, \boldsymbol{\Theta})\mathsf{P}(\text{NM}_1|\boldsymbol{\Theta}).
\end{aligned}$$

We assume that the probability of the non-Mendelian model is 1.5×10^{-6} (as in the joint HMM). Under the Mendelian model ($\text{NM}_1 = 1$), the conditional probability for the offspring copy number is given by previously published tabled probabilities (Supplementary Table 1, [5]). The tabled probabilities in Wang *et al.* are a function of the parameter a, corresponding to the probability of the less likely chromosome configuration accounting for the total copy number. The default in the joint HMM is $a = 0.0009$, and we have adopted the same default. (Wang *et al.* assume that there are two *chromosome-specific* configurations for a given total copy number and that the other configurations have negligible probability.)

For segments $l > 1$, the probability of the trio copy number is conditional on the copy number of the previous segment and can be factored as in equation (4) (Section Results and discussion) into terms that include the joint probability of offspring copy number at adjacent segments, a transition probability for the parental copy numbers, and the marginal probability for the parental copy number of the previous segment. Again, we average the conditional probability for the offspring copy number over latent, binary indicators for Mendelian transmission. Here, three scenarios are possible depending on whether the adjacent segments are both Mendelian, only one is Mendelian, or both are non-Mendelian. We leave the details of the derivation of the joint probability to the original paper [5]. We use $\frac{1}{2}$ for the transition probability when the copy number states are the same and $\frac{1}{8}$ otherwise. Similarly, the transition probability for that both segments are Mendelian or both are non-Mendelian is $\frac{1}{2}$ and $\frac{1}{8}$ when only one segment is Mendelian. When only one segment is Mendelian, we use previously published probabilities of Mendelian transmission (Supplementary Table 1, [5]) and assume that any of the states are equally probable for the non-Mendelian segment. When both segments are Mendelian, we execute a look up of tabled probabilities generated from PennCNV's

source code [6]. PennCNV assumes two chromosome-specific copy number configurations are predominant for Mendelian transmission (Table 3 in [5]) and that the less likely of the two configuration occurs with probability 0.0009. We adopt the same defaults. Finally, we assume that any of the copy number states at segment $l - 1$ are equally probable for the parents.

PennCNV annotation for trio copy number states

PennCNV concisely annotates the trio copy number state for a genomic interval as $s_F s_M s_O$. For example, the trio state '332' corresponds to a de novo hemizygous deletion in the offspring ($s_O = 2$) as both parents are diploid ($s_F = s_M = 3$). We adopt the same annotation of the state calls in the supplemental figures.

For some intervals, PennCNV assigns multiple states. For example, the hyphenated state "332-232" indicates that algorithm infers that the offspring has a hemizygous deletion for which part is de novo ("332") and part is transmitted from the father ("232"). We adjudicate multi-state PennCNV calls in our assessment of concordance with MinimumDistance as follows. For state '332', we regarded the inferences from PennCNV and MinimumDistance as discordant if any of the multiple states assigned by PennCNV included the call '332' and none of the overlapping MinimumDistance calls were '332'. Conversely, if any of the overlapping MinimumDistance calls were '332' we regarded the region as concordant.

Empirical estimation of simulation parameters in the oral cleft study

We assessed how the simulated variance and correlations of the log R ratios for a trio reflect the empirically estimated log R ratio variance and correlations in the oral cleft trios. Nearly 81% of samples in the oral cleft study had a MAD less than 0.2. Moderately high ($\sigma_r = 0.25$) and high ($\sigma_r = 0.30$) standard deviations in the simulation comprise 9% and 4% of the oral cleft trios, respectively (rows 3 and 4 of Figure2). The remaining six percent of the trios had standard deviations exceeding 0.30 and were subsequently excluded by quality control (see Methods). As an estimate of the log R ratio correlation for members in a trio, we subtracted the CBS segment means from the autosomal log R ratios to obtain a 600,470 x 3 matrix of demeaned log R ratios. We calculated the maximum of the father-mother, mother-offspring, and father-offspring log R ratio correlations. The percentage of trios with a maximum correlation in the intervals (0.1, 0.3] and (0.3, 1.0] were 29% and 62% respectively (columns 2 and 3 of Figure 2b). Fewer than 10% of the trios had correlations less than 0.10 (column 1, Figure 2b).

R environment and software versions

- R version 2.15.1 (2012-06-22), `x86_64-apple-darwin11.4.0`
- Locale: `en_US.US-ASCII/en_US.US-ASCII/en_US.US-ASCII/C/en_US.US-ASCII/en_US.US-ASCII`
- Base packages: base, datasets, graphics, grDevices, methods, stats, tools, utils
- Other packages: Biobase 2.18.0, BiocGenerics 0.4.0, bit 1.1-8, CleftExperimentData 1.1.2, ff 2.2-7, foreach 1.4.0, IRanges 1.16.2, MinimumDistance 1.2.2, oligoClasses 1.20.0
- Loaded via a namespace (and not attached): affyio 1.26.0, annotate 1.36.0, AnnotationDbi 1.20.1, BiocInstaller 1.8.3, Biostrings 2.26.2, codetools 0.2-8, crlmm 1.16.3, DBI 0.2-5, DNAcopy 1.32.0, ellipse 0.3-7, genefilter 1.40.0, GenomicRanges 1.10.2, grid 2.15.1, iterators 1.0.6, lattice 0.20-10, msm 1.1.3, mvtnorm 0.9-9992, parallel 2.15.1, preprocessCore 1.20.0, RSQLite 0.11.2, SNPchip 2.4.0, splines 2.15.1, stats4 2.15.1, survival 2.36-14, VanillaICE 1.20.3, XML 3.95-0.1, xtable 1.7-0, zlibbioc 1.4.0

References

1. Baum L, Petrie T, Soules G, Weiss N: **A maximization technique occurring in the statistical analysis of probabilistic functions of Markov chains**. *Ann. Math. Statist.* 1970, **41**:164–171.
2. Scharpf RB, Parmigiani G, Pevsner J, Ruczinski I: **Hidden Markov models for the assessment of chromosomal alterations using high-throughput SNP arrays**. *Annals of Applied Statistics* 2008, **2**(2):687–713, [http://dx.doi.org/10.1214/07-AOAS155].
3. **The Bioconductor Project** [http://www.bioconductor.org].
4. Viterbi A: **Error bounds for convolution codes and an asymptotically optimal decoding algorithm**. *IEEE Transactions on Information Theory* 1967, **13(2)**:260–269.
5. Wang K, Chen Z, Tadesse MG, Glessner J, Grant SFA, Hakonarson H, Bucan M, Li M: **Modeling genetic inheritance of copy number variations.** *Nucleic Acids Res* 2008, **36**(21):e138, [http://dx.doi.org/10.1093/nar/gkn641].
6. **PennCNV manual** [http://www.openbioinformatics.org/penncnv].
