# Supplementary material for: Fast detection of de novo copy number variants from SNP arrays for case-parent trios
Source: BMC Bioinformatics. 2012 Dec 12;13:330. doi: 10.1186/1471-2105-13-330 (PMC3576329; doi:10.1186/1471-2105-13-330)
Supplement: Additional file 2 — Supplementary figures. Supporting figures. [file 1471-2105-13-330-S2.pdf]

Supplementary Figures

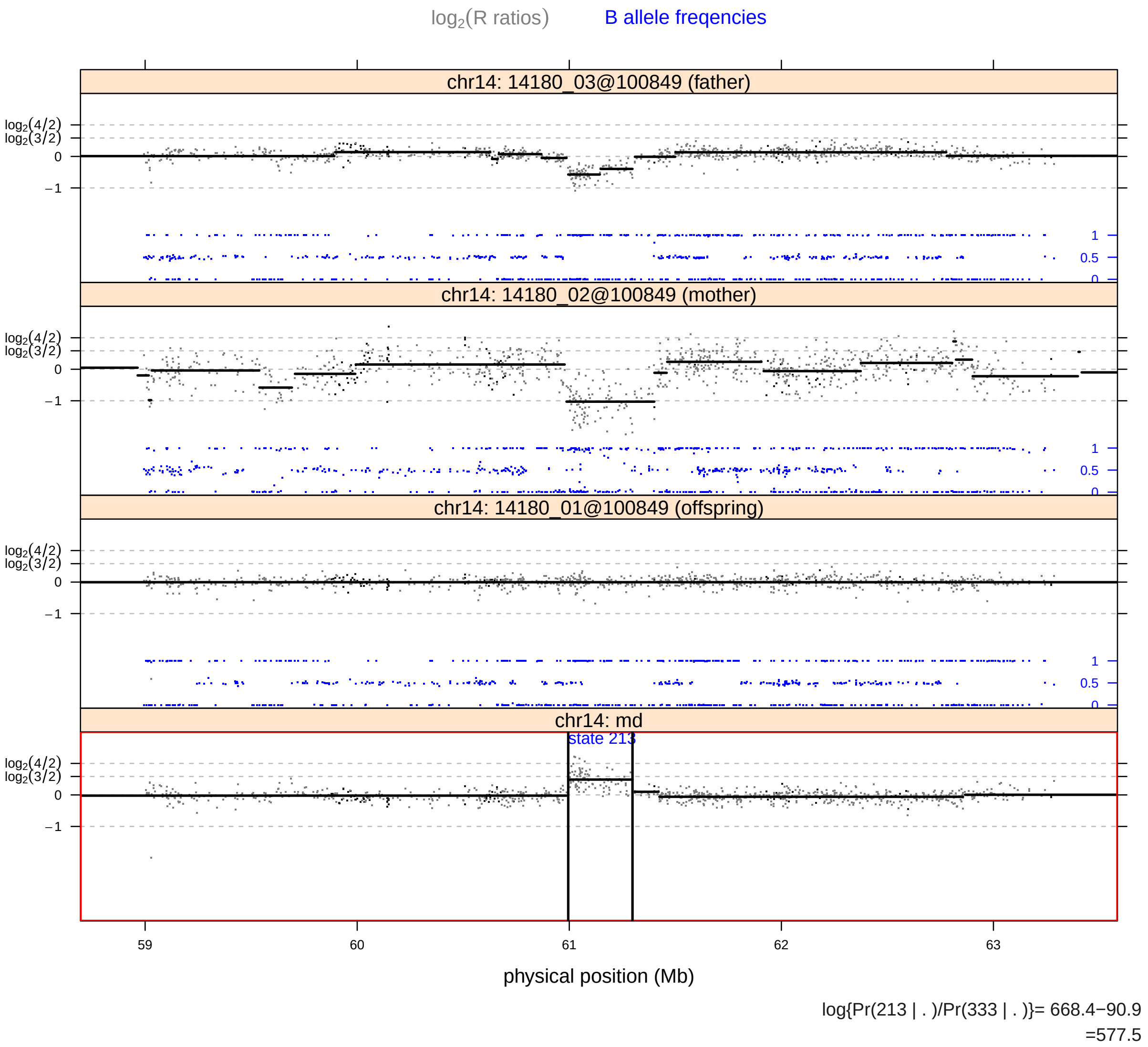

Figure S1: The first three panels from the top plot physical position versus the log R ratios (gray) and B allele frequencies (blue) for the father, mother, and offspring. The bottom panel plots the minimum distance. Overlaying the log R ratios and the minimum distance in panels 1-4 is the CBS segmentation (black line segments). Both parents have an apparent deletion at the locus bound by the vertical black lines, while the offspring has normal copy number as indicated by B allele frequencies and log R ratios near zero in panel 3. While the positive minimum distance in panel 4 suggests a potentially de novo copy number gain (bottom panel), the maximum a posteriori estimate is "213" corresponding to a hemizygous deletion in the father (state 2), a possible homozygous deletion in the mother (state 1), and diploid copy number in the offspring (state 3). Such a trio state represents a gain relative to the parental copy numbers, but we do not regard diploid autosomal copy numbers as CNVs.

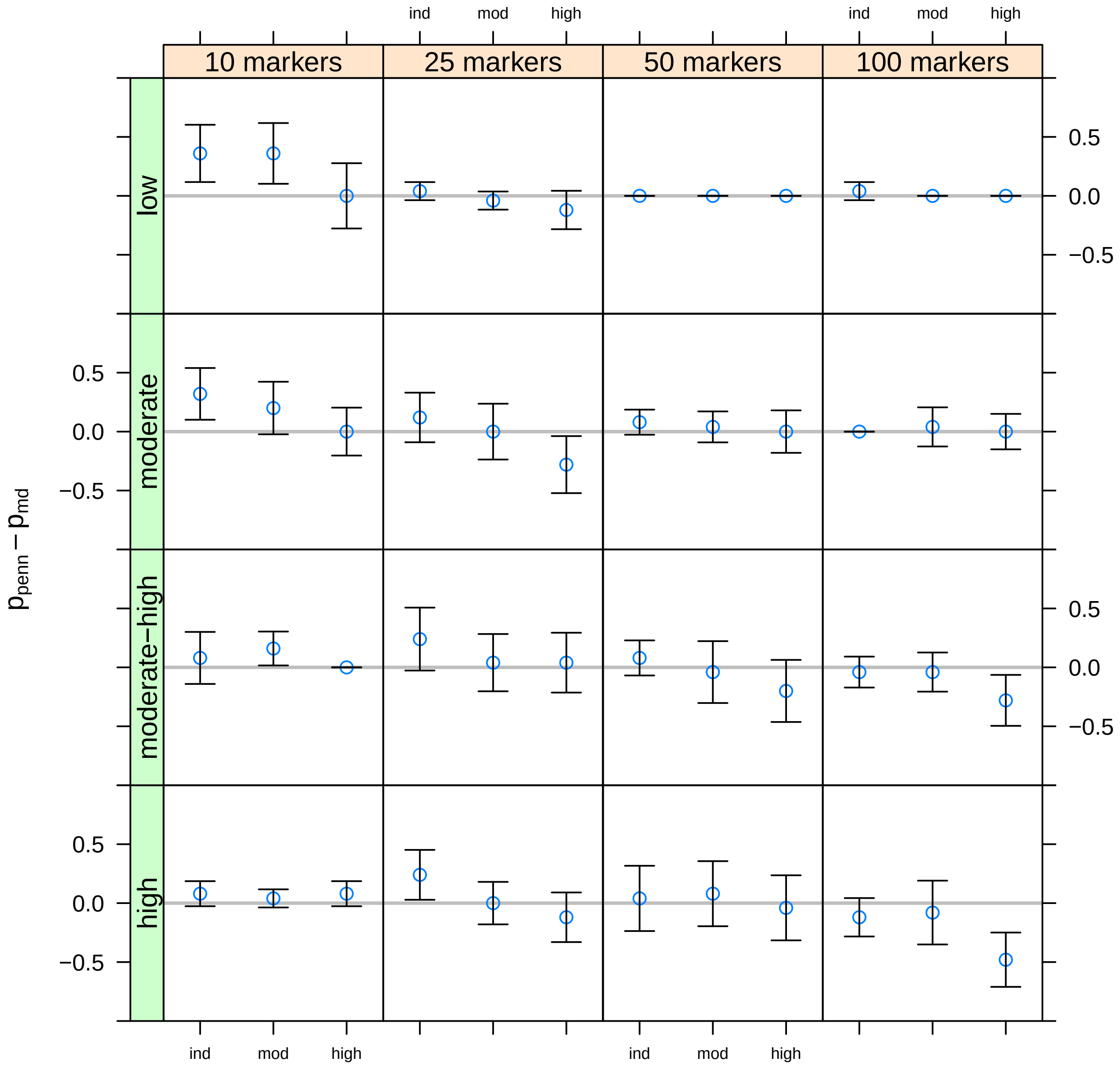

Figure S2: For this plot, we called a simulated de novo feature a FN if more than 50% of the markers in the feature were incorrectly called. The three boxplots in each panel correspond to different levels of correlation of the log R ratios for the members in a trios. In particular, we simulated uncorrelated log R ratios (ind), moderate correlation (0.2), and high correlation (0.5). Columns of the 4×3 grid indicate the coverage of the simulated de novo features and rows indicate the level of the log R ratio variance (see Methods). The y-axis in each panel plots the difference in the mean proportion of FN calls from 25 synthetic chromosomes (circle) with error bars denoting $\pm$ two standard errors. The fraction of de novo deletions that were FNs for features with 25 or more markers was similar. Most of the oral cleft trios (81 percent) have empirically estimated standard deviations in the low-moderate range (rows 1 and 2). For features with only 10 markers (column 1), MinimumDistance tended to have fewer FNs.

De novo hemizygous deletions called only by PennCNV

Interpretation of Figures S3–S7. We ranked PennCNV de novo hemizygous deletion calls by coverage. Of the top 100 de novo PennCNV calls, 47 were discovered only by PennCNV. Examples of the PennCNV-only calls are plotted in Figures S3 – S7. Each figure plots the log R ratios of SNPs (gray) and nonpolymorphic markers (dark gray) as well as the B allele frequencies (blue) for the trio in the top 3 panels. The bottom panel plots the minimum distance (md). Overlaying the log R ratios and the minimum distance is the segmentation from the CBS algorithm indicated by the black line segments. The orange vertical lines denote the breakpoints from PennCNV. The posterior probability for the maximum a posteriori estimate of the region bounded by the orange lines is proportional to the reported numerator, and the posterior probability of diploid copy number is proportional to the reported denominator (bottom right margin). Note that the region bound by the orange lines may differ substantially from the breakpoints identified by the segmentation of the minimum distance. As a consequence, the maximum a posteriori estimate of the PennCNV segment may differ from the maximum a posteriori estimate of the minimum distance segment spanning the PennCNV call (bottom left margin). When the maximum a posteriori estimate is the diploid state (e.g., see Figure S3), the log ratio of posterior probabilities is zero.

Some of these regions have multiple states assigned by PennCNV. For example, the segment in Figure S3 has the call '332-232', indicating that the region contains a de novo hemizygous deletion as well as a possible inherited hemizygous deletion from the father. The genomic location at which the father transitions to a hemizygous deletion for the locus is not specified by PennCNV.

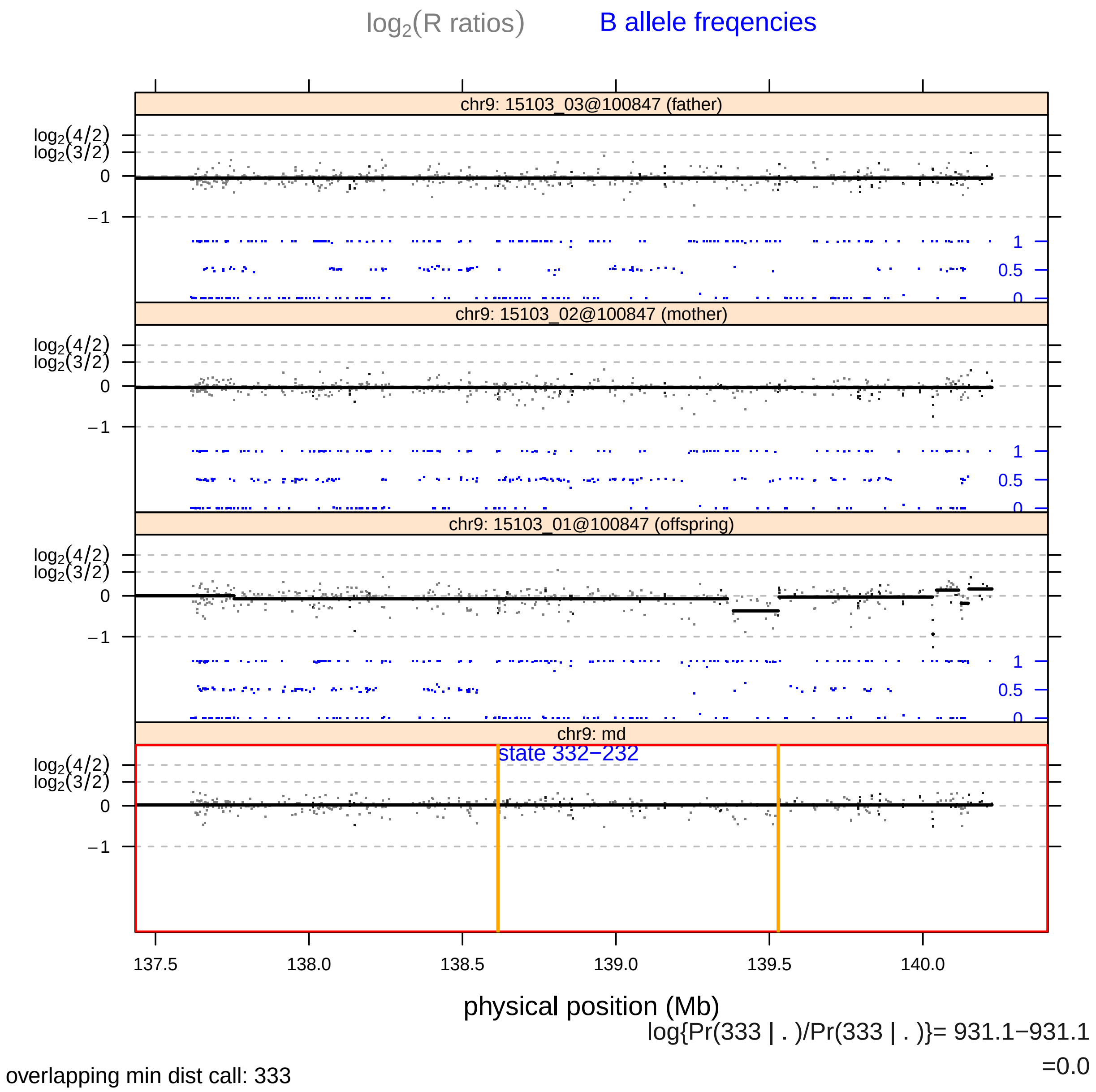

Figure S3: Likely a false positive by PennCNV for '332'. PennCNV assigns the hyphenated state '332-232' to the region demarcated by the orange vertical lines. The '232' region called by PennCNV likely corresponds to a small region at 139.5 Mb. The entire plotted region has a minimum distance near zero. The breakpoints from minimum distance lie beyond the plotted region and are placed at the margins. The a posteriori classification for minimum distance is based on a much larger region than the locus demarcated by orange lines. As most of the broader region is consistent with normal copy number, the minimum distance classification is '333'.

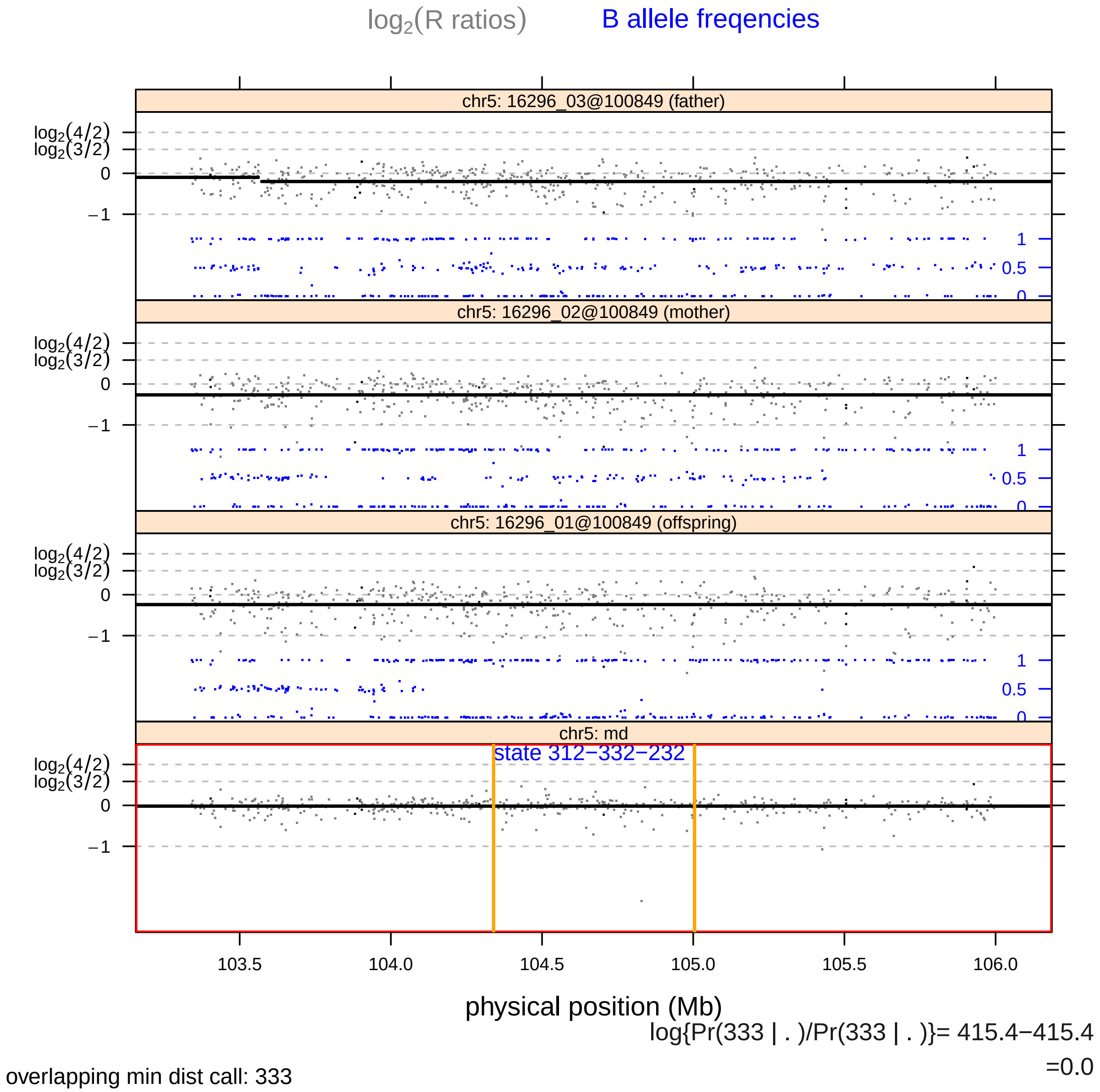

Figure S4: A false positive induced by a genomic wave that is present in all members of the trio. The minimum distance has a much smaller variance and is centered at zero, suggesting no difference in copy number between parents and offspring.

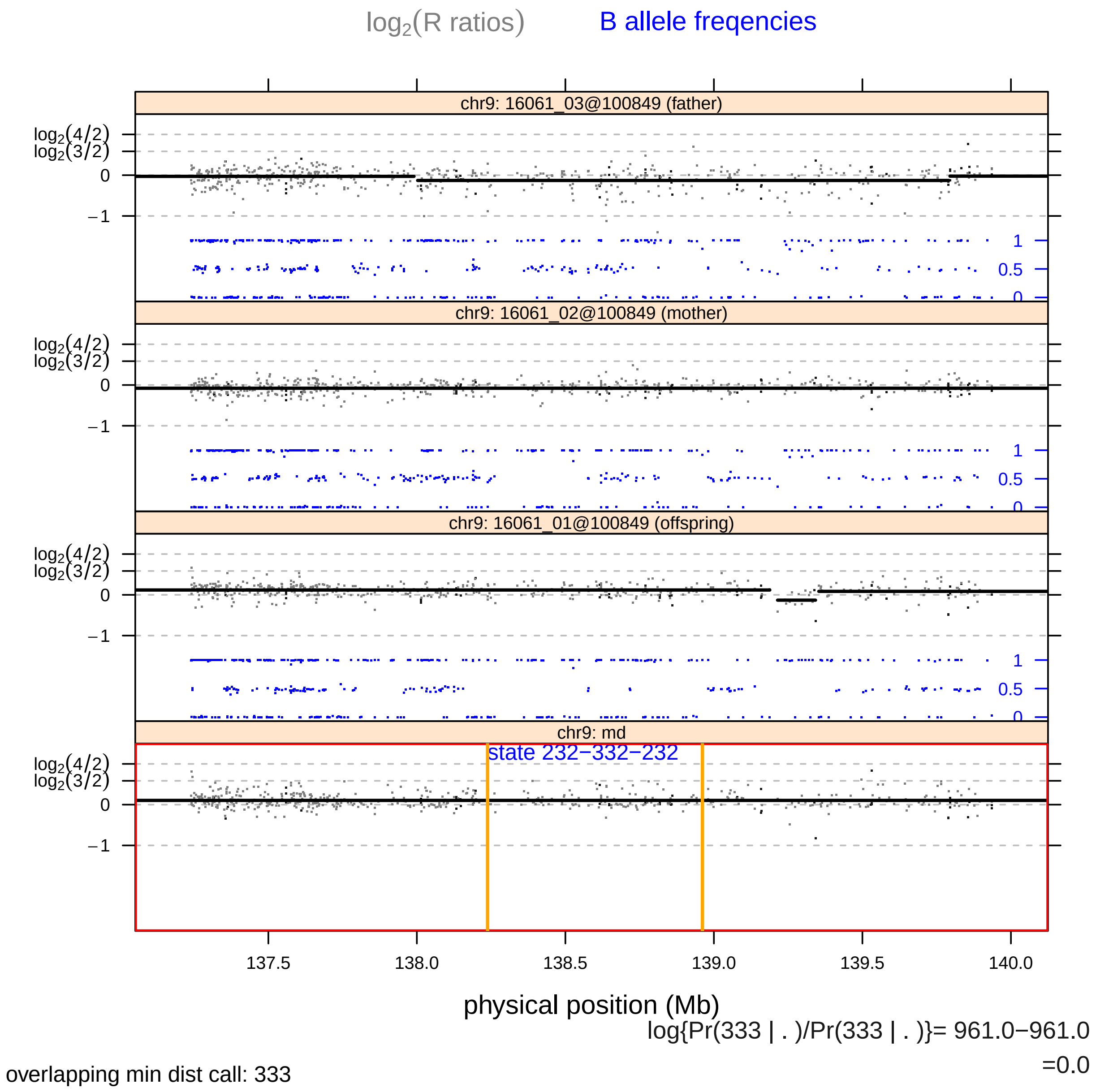

Figure S5: The de novo hemizygous deletion called by PennCNV is likely a false positive.

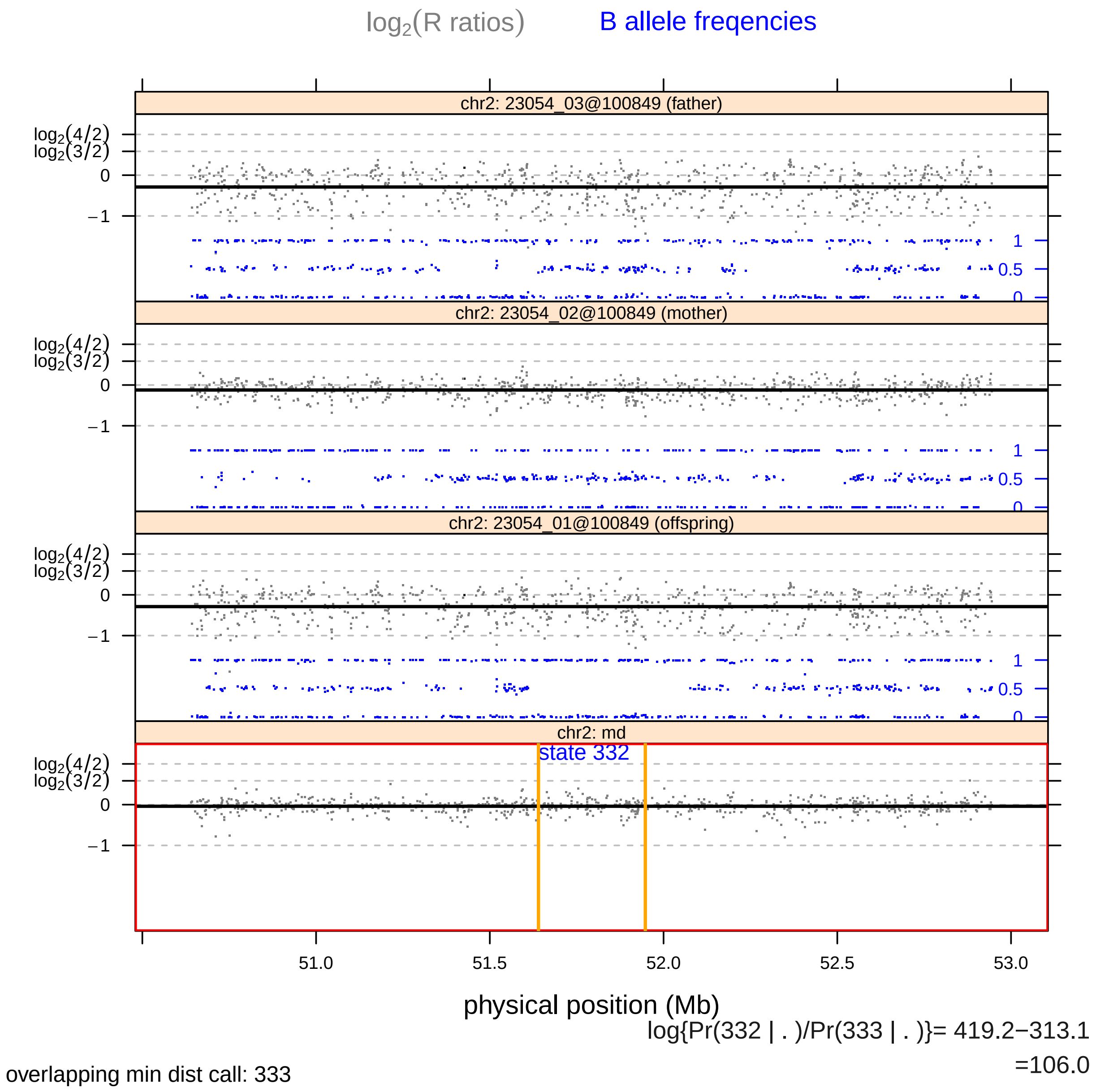

Figure S6: Likely a false positive induced by a genomic wave present in the father and offspring.

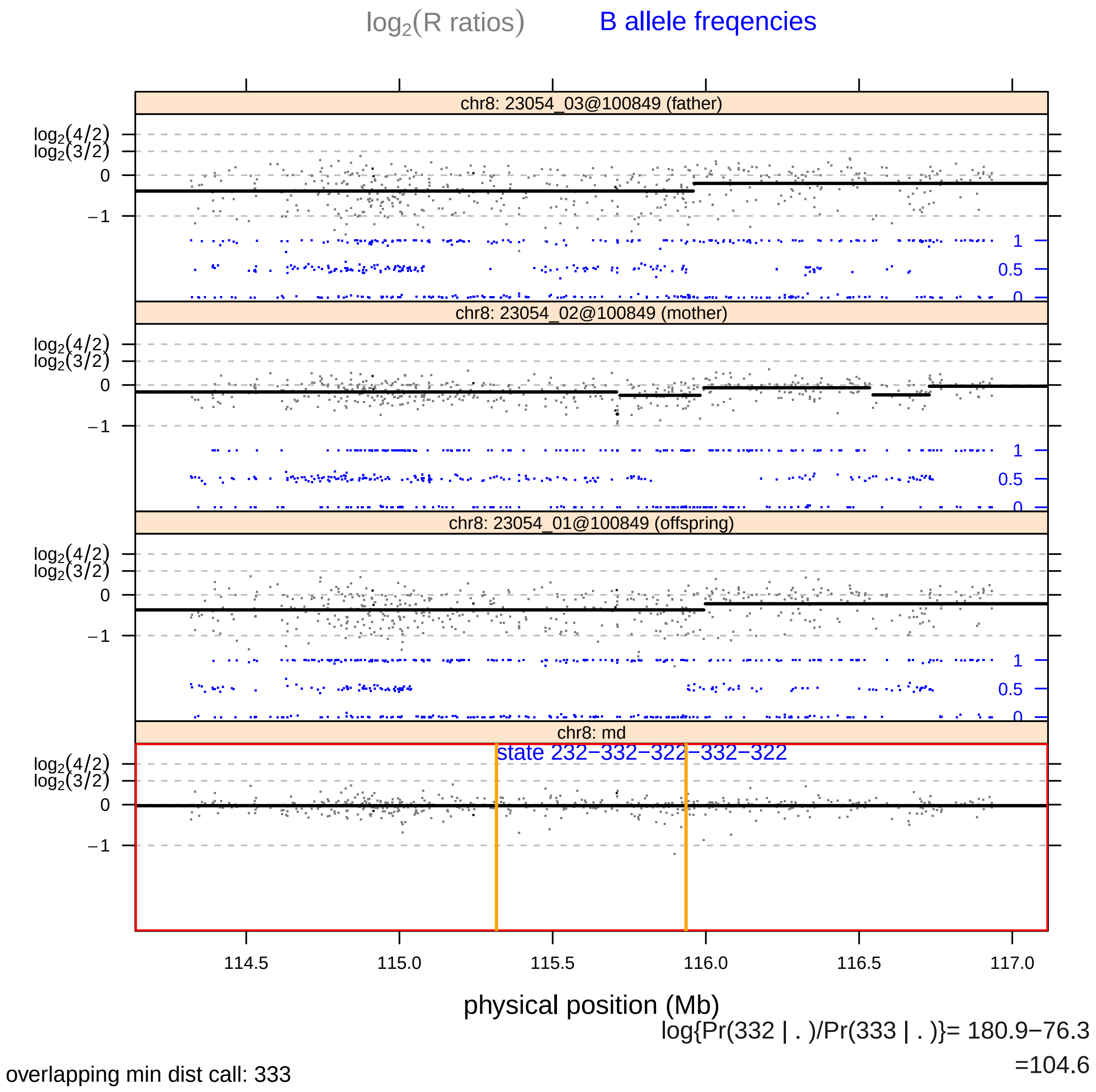

Figure S7: A false positive induced by a genomic wave that appears similar in the father and offspring.

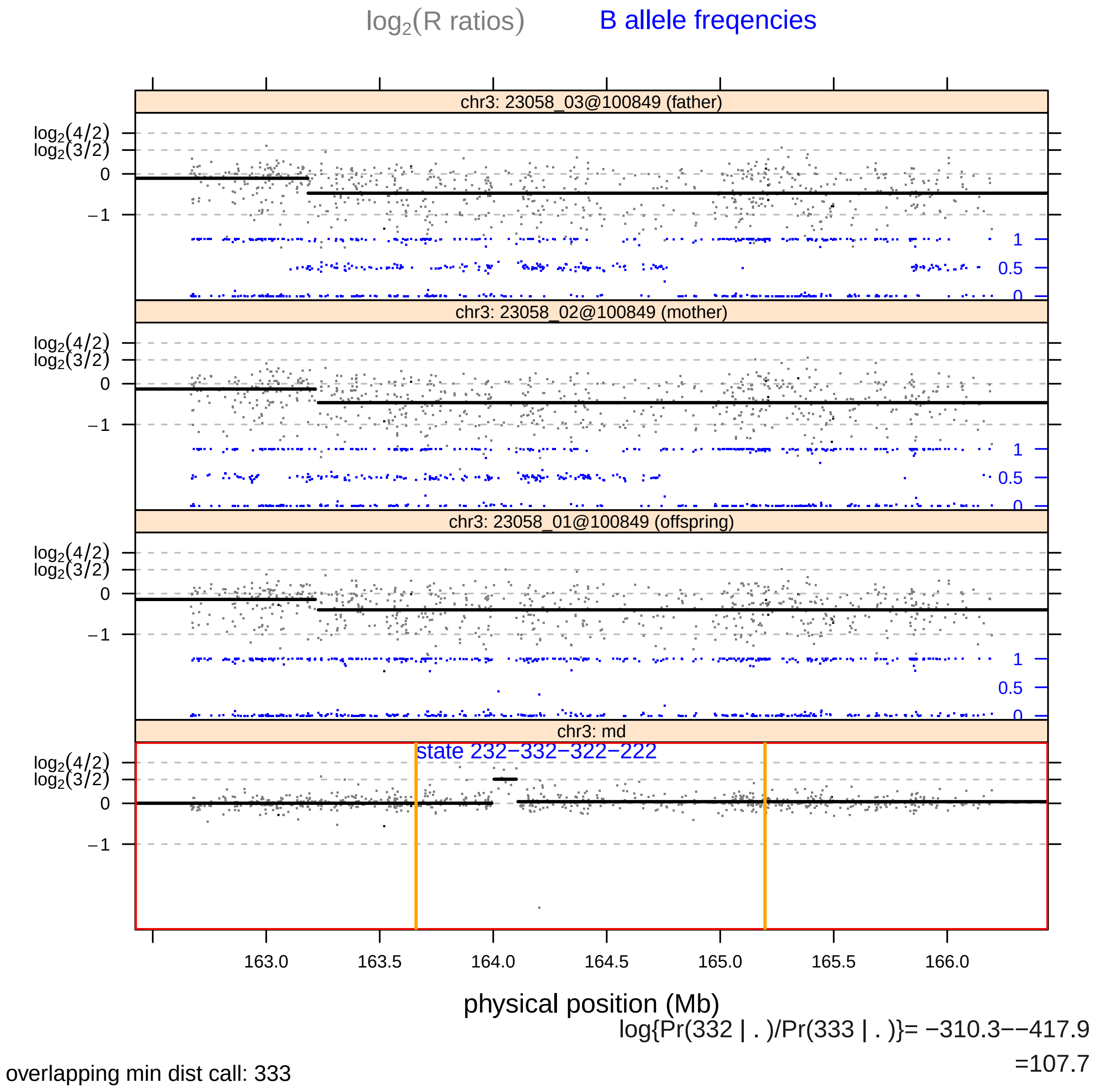

Figure S8: Likely a false positive by PennCNV for '332'. The offspring log R ratios have a high level of noise.

De novo hemizygous deletions called only by MinimumDistance

Interpretation of Figures S9–S14 .

We ranked the de novo hemizygous deletions called by MinimumDistance by coverage. Examples of de novo calls with high coverage that were called by MinimumDistance but not called by PennCNV are plotted in Figures S9–S14. The PennCNV call spanning the region is indicated in the bottom left margin.

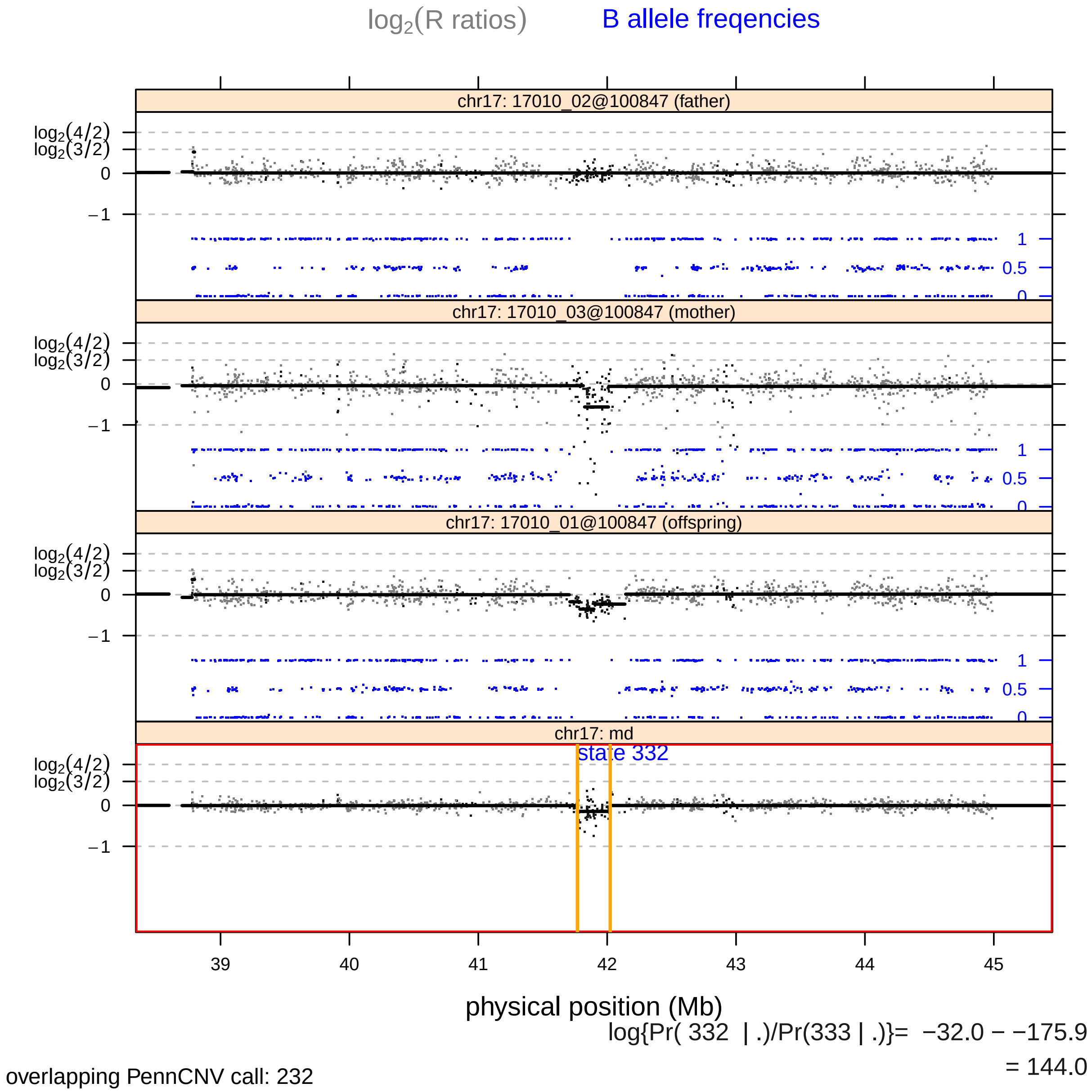

Figure S9: This is a complex CNV that appears to be mostly inherited from the mother. However, the hemizygous deletion appears larger in the offspring and may be comprised of both de novo and maternally transmitted copy number. Multi-state calls with MinimumDistance are not permitted.

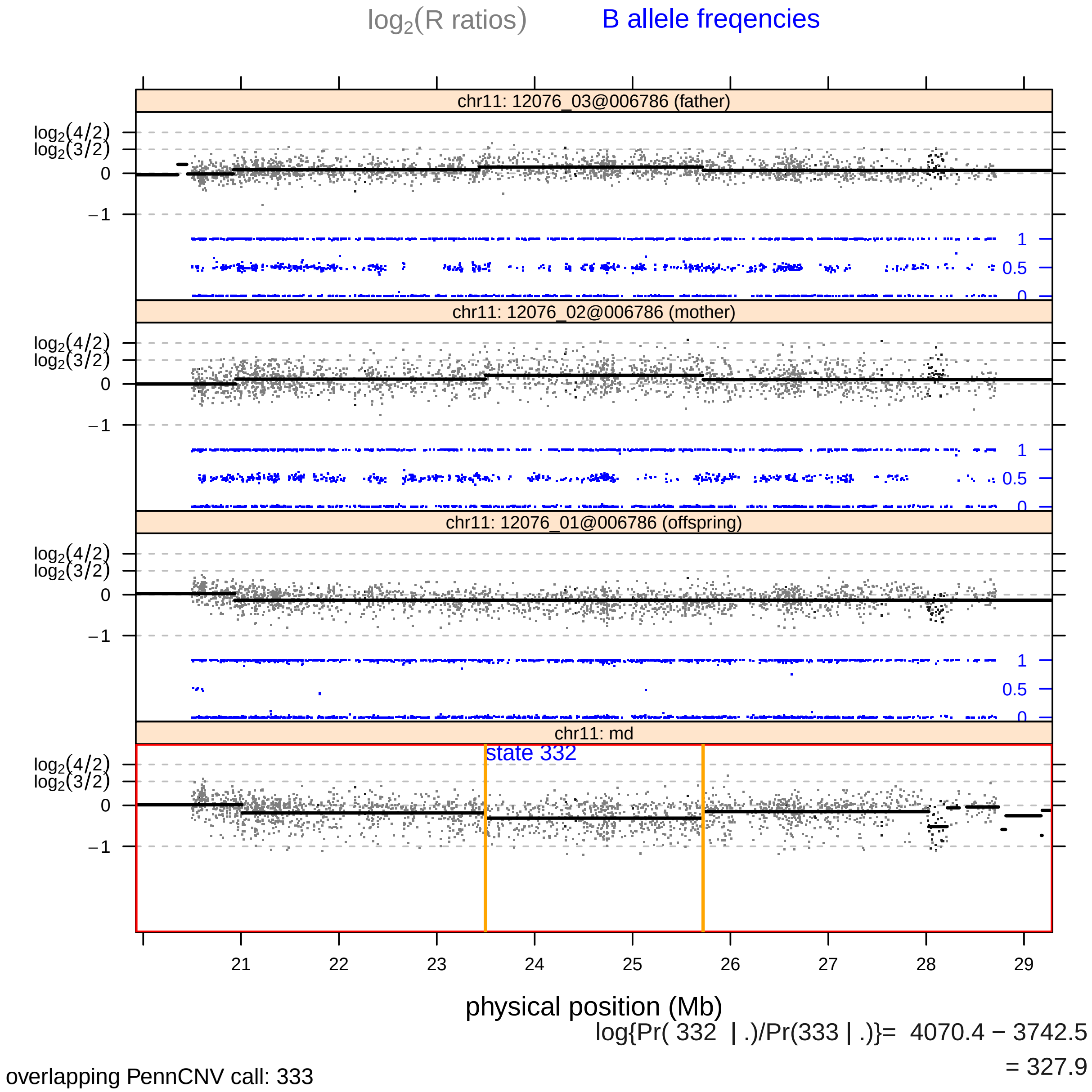

Figure S10: This apparent false positive appears to be induced by a genomic wave that is slightly inverted in the parents compared to the offspring. Future versions of MinimumDistance may flag wave inversions, or apply methods to adjust for such artifacts.

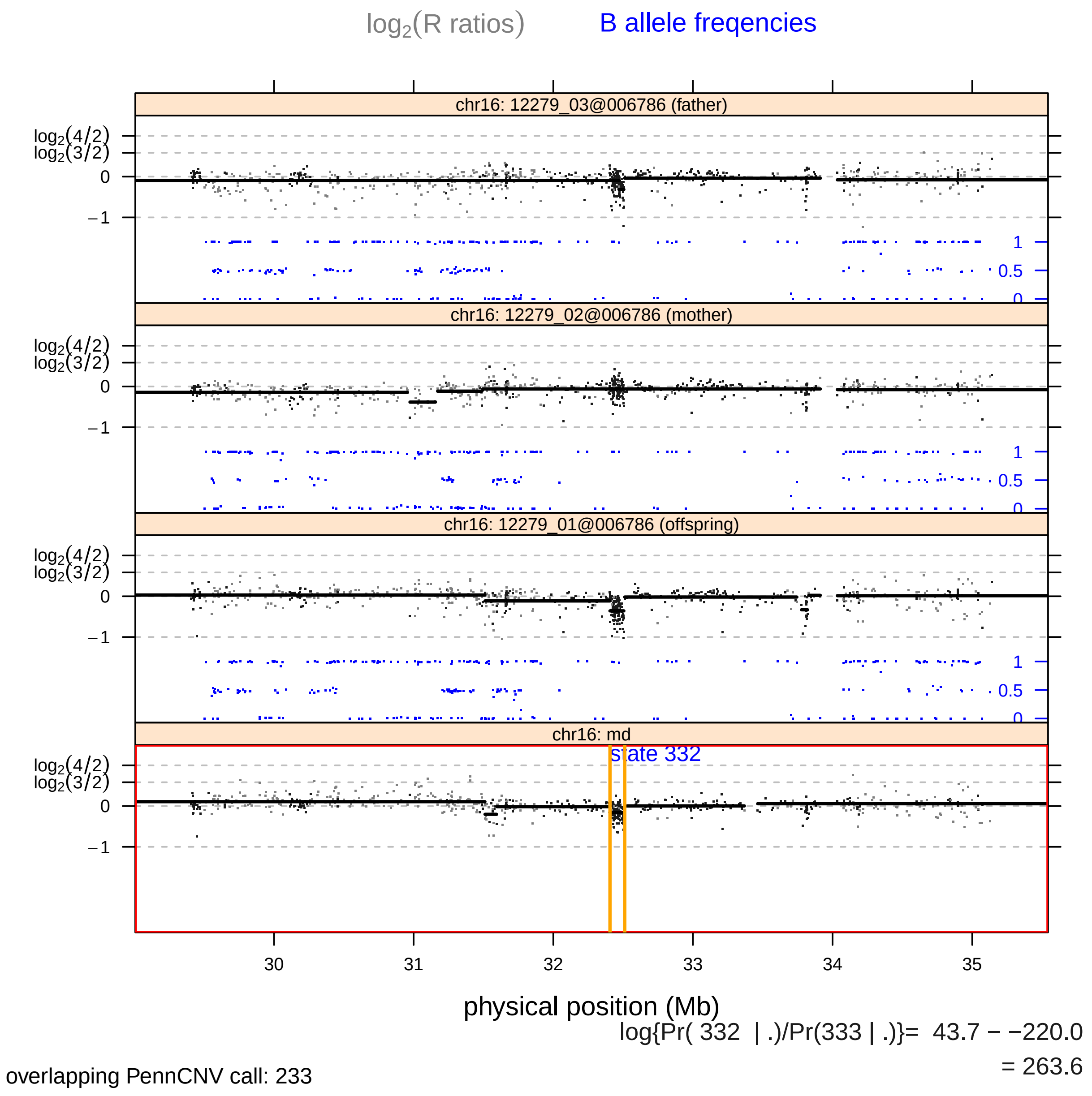

Figure S11: The called region is covered mostly by nonpolymorphic markers. Likely a false positive for ‘332’ called by minimum distance as the father also has negative log R ratios in this region, though differing in magnitude.

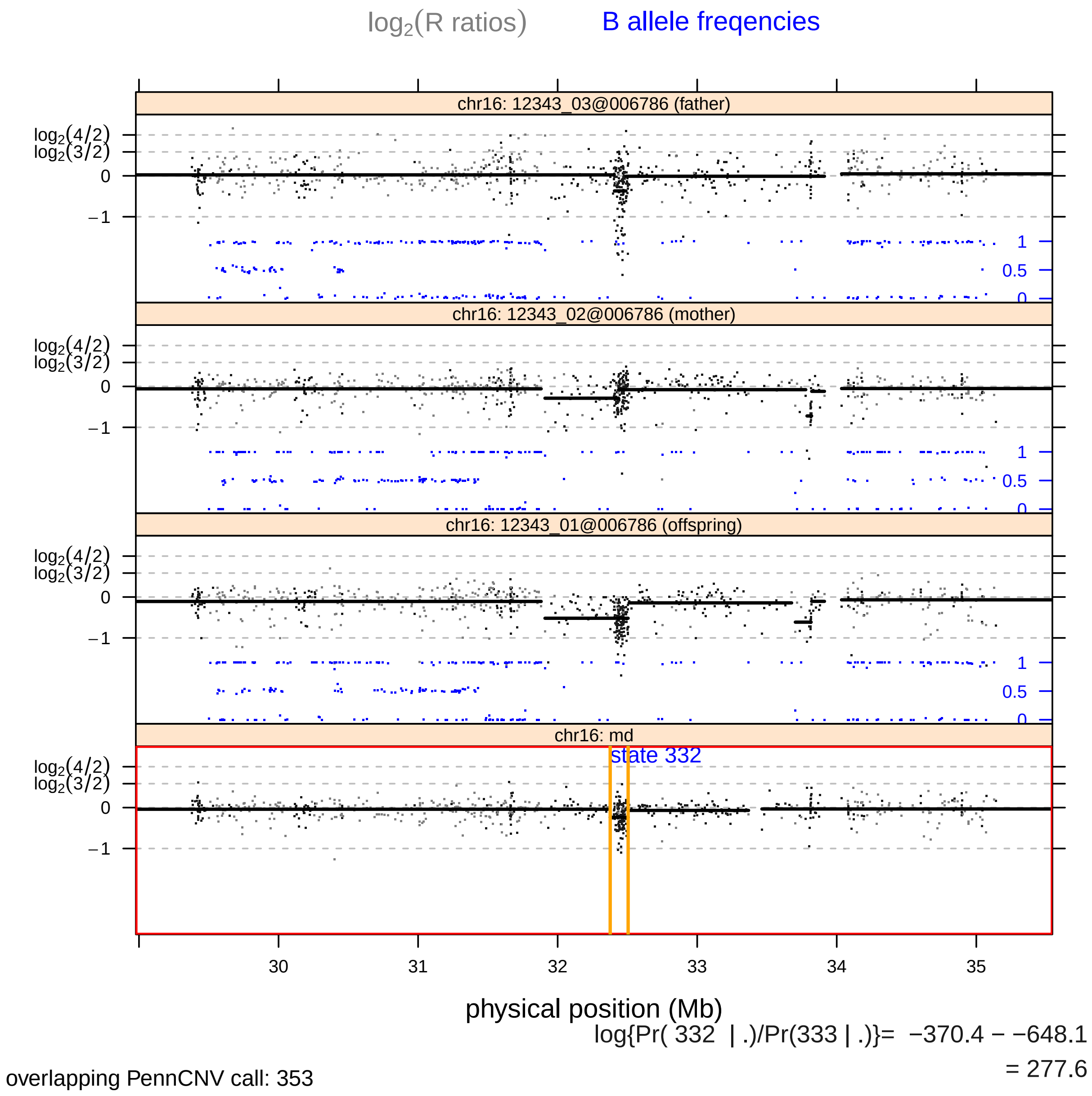

Figure S12: Similar interpretation as Figures S9 and S11.

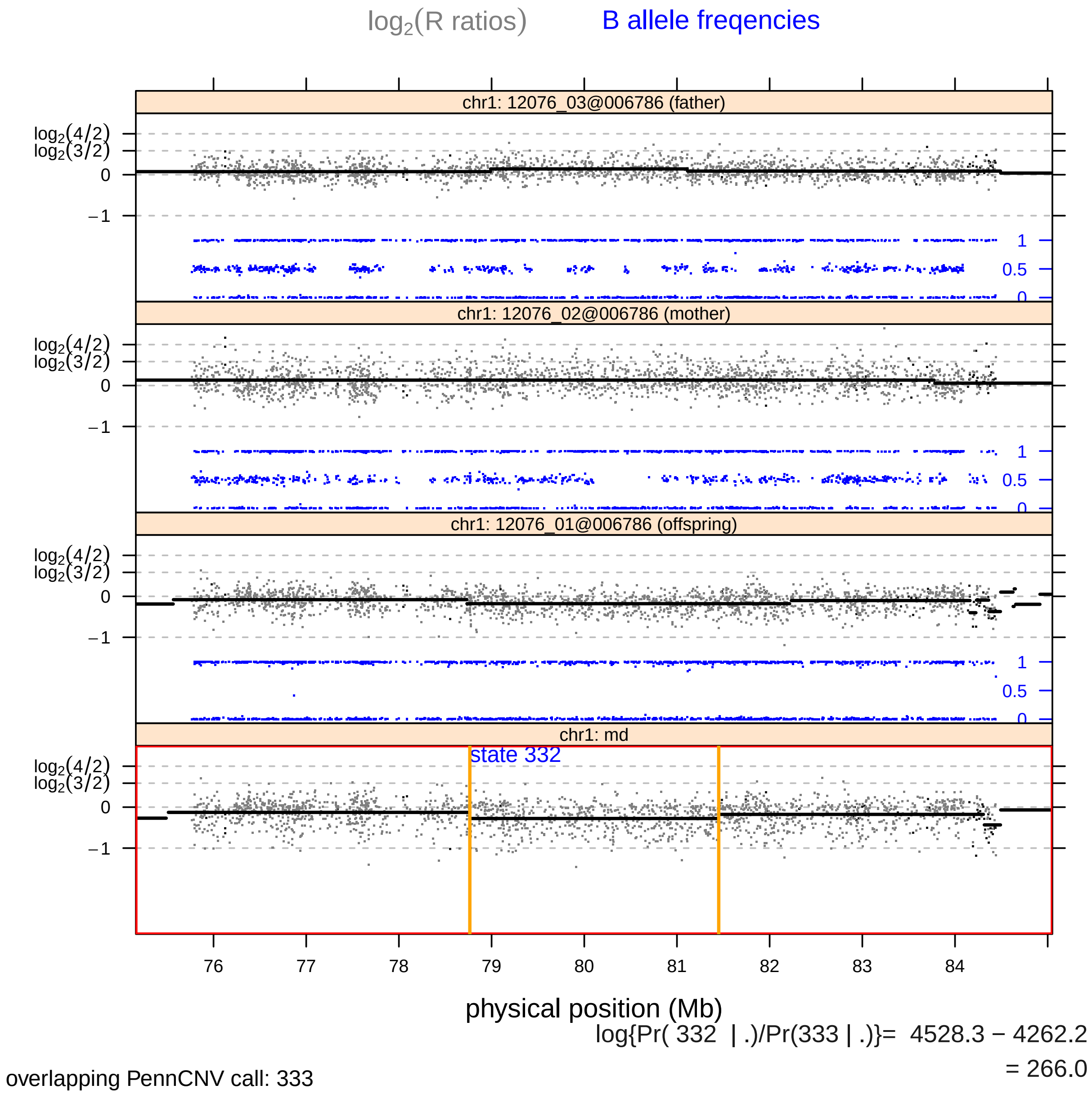

Figure S13: This is the same sample as in Figure S10 – a slightly inverted genomic wave in the parents and offspring results in a false positive.

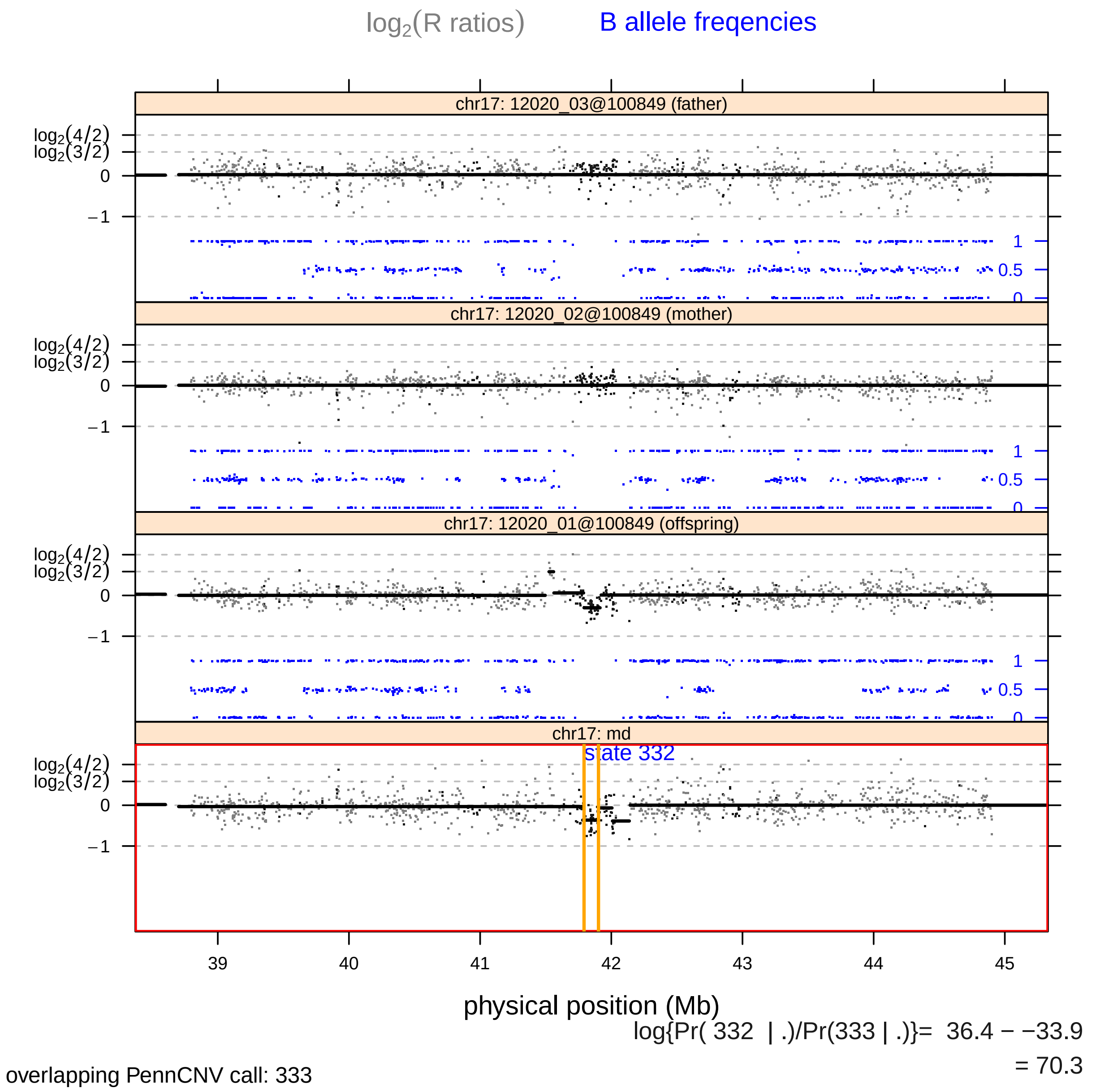

Figure S14: The data is consistent with a de novo deletion in the offspring called by MinimumDistance that was undetected by by PennCNV. However, the region is covered only by nonpolymorphic markers which carry less information than SNPs (no information regarding allele frequencies). Experimental validation is needed.

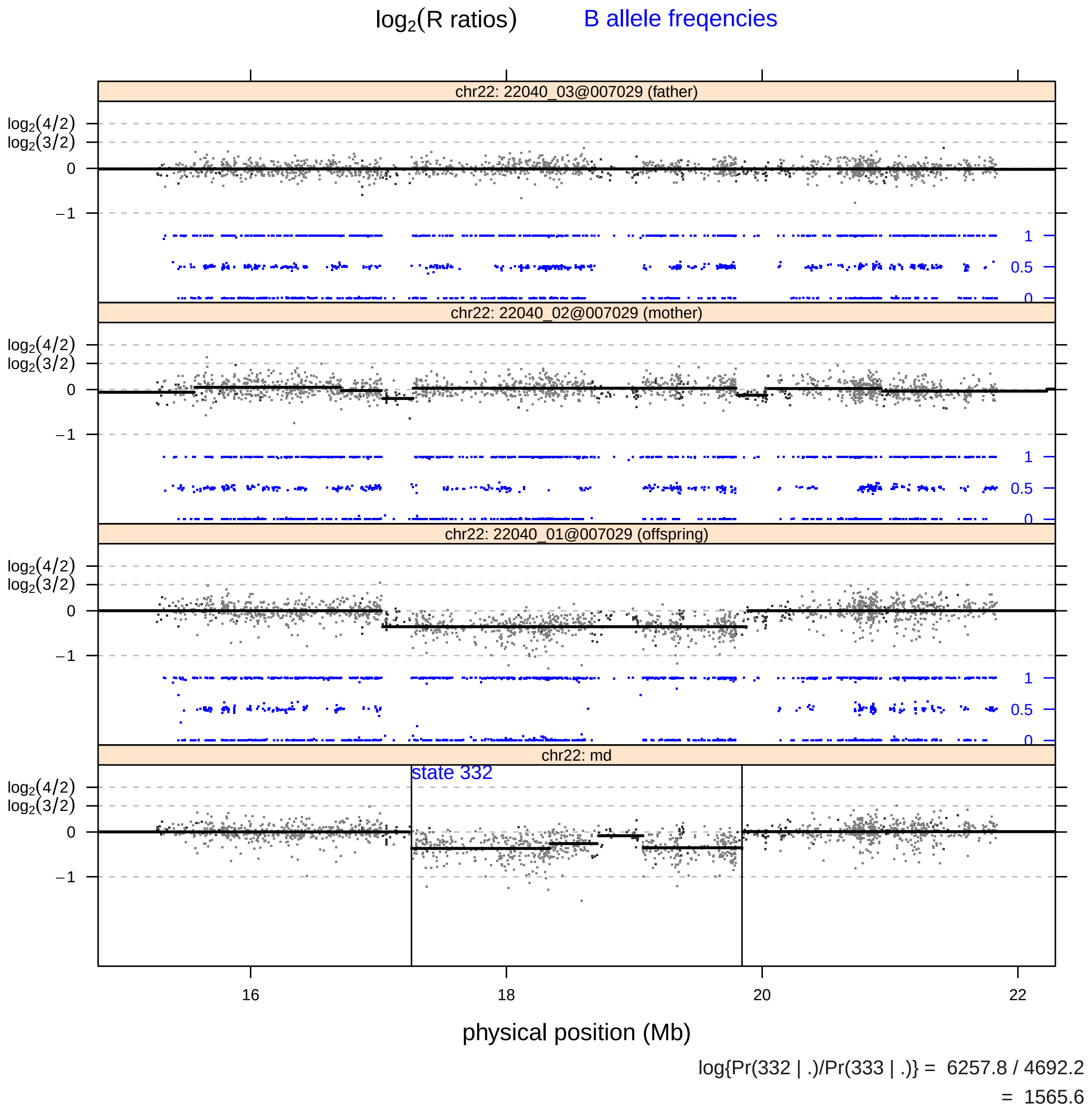

Figure S15: Log R ratios (gray) and B allele frequencies (blue) for father, mother, and offspring for an apparent de novo hemizygous deletion occurring in the DiGeorge critical region (panels 1-3). The minimum distance is plotted in panel 4. An interval containing a called de novo hemizygous deletion is demarcated by the vertical black lines. The CBS segmentation is indicated by the over-plotted black line segments. The log ratio of the posterior probability for the de novo hemizygous state and diploid states are provided in the bottom right margin.

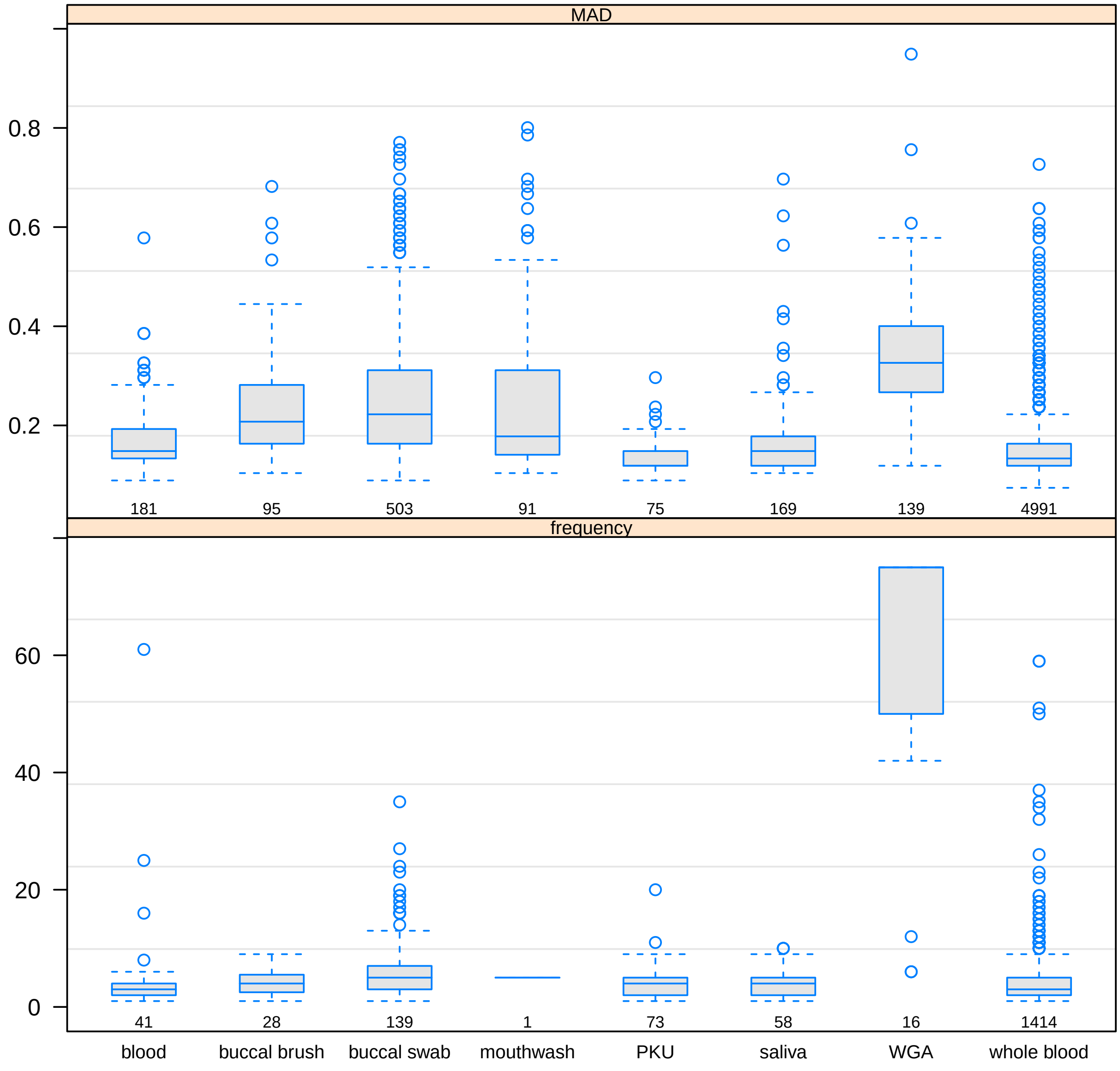

Figure S16: The log R ratio MAD (top) and frequency of de novo CNVs (bottom) stratified by DNA source. Samples with DNA source that was whole genome amplified (WGA) and samples with log R ratio MAD exceeding 0.3 were subsequently excluded. The frequency of de novo events in the whole genome amplified (WGA) samples ranged from 0 to 375. Samples with more than 75 de novo events are thresholded at 75 in the bottom panel.
